# Chromosome-level reference genome of *Vitis piasezkii* var. *pagnucii* provides insights into a new locus of resistance to grapevine powdery mildew

**DOI:** 10.1093/hr/uhaf146

**Published:** 2025-06-10

**Authors:** Liang Zhao, Yang Hu, Qian-Yu Ji, Li-Xue Gong, Meng-Jiao Lu, Xue-Na Yu, Zhuo-Shuai Jin, Min Zhou, Xue-Lei Dai, Shun-Yuan Xiao, Yu Jiang, Ying-Qiang Wen

**Affiliations:** State Key Laboratory of Crop Stress Resistance and High-Efficiency Production, College of Horticulture, Northwest A&F University, Yangling Shaanxi 712100, China; Key Laboratory of Horticultural Plant Biology and Germplasm Innovation in Northwest China, Ministry of Agriculture and Rural Affairs, Yangling Shaanxi 712100, China; State Key Laboratory of Crop Stress Resistance and High-Efficiency Production, College of Horticulture, Northwest A&F University, Yangling Shaanxi 712100, China; Key Laboratory of Horticultural Plant Biology and Germplasm Innovation in Northwest China, Ministry of Agriculture and Rural Affairs, Yangling Shaanxi 712100, China; State Key Laboratory of Crop Stress Resistance and High-Efficiency Production, College of Horticulture, Northwest A&F University, Yangling Shaanxi 712100, China; Key Laboratory of Horticultural Plant Biology and Germplasm Innovation in Northwest China, Ministry of Agriculture and Rural Affairs, Yangling Shaanxi 712100, China; State Key Laboratory of Crop Stress Resistance and High-Efficiency Production, College of Horticulture, Northwest A&F University, Yangling Shaanxi 712100, China; Key Laboratory of Horticultural Plant Biology and Germplasm Innovation in Northwest China, Ministry of Agriculture and Rural Affairs, Yangling Shaanxi 712100, China; State Key Laboratory of Crop Stress Resistance and High-Efficiency Production, College of Horticulture, Northwest A&F University, Yangling Shaanxi 712100, China; Key Laboratory of Horticultural Plant Biology and Germplasm Innovation in Northwest China, Ministry of Agriculture and Rural Affairs, Yangling Shaanxi 712100, China; State Key Laboratory of Crop Stress Resistance and High-Efficiency Production, College of Horticulture, Northwest A&F University, Yangling Shaanxi 712100, China; Key Laboratory of Horticultural Plant Biology and Germplasm Innovation in Northwest China, Ministry of Agriculture and Rural Affairs, Yangling Shaanxi 712100, China; State Key Laboratory of Crop Stress Resistance and High-Efficiency Production, College of Horticulture, Northwest A&F University, Yangling Shaanxi 712100, China; Key Laboratory of Horticultural Plant Biology and Germplasm Innovation in Northwest China, Ministry of Agriculture and Rural Affairs, Yangling Shaanxi 712100, China; State Key Laboratory of Crop Stress Resistance and High-Efficiency Production, College of Horticulture, Northwest A&F University, Yangling Shaanxi 712100, China; Key Laboratory of Horticultural Plant Biology and Germplasm Innovation in Northwest China, Ministry of Agriculture and Rural Affairs, Yangling Shaanxi 712100, China; Key Laboratory of Animal Genetics, Breeding and Reproduction of Shaanxi Province, College of Animal Science and Technology, Northwest A&F University, Yangling Shaanxi 712100, China; Department of Plant Science and Landscape Architecture, Institute for Bioscience and Biotechnology Research, University of Maryland, Rockville, MD, USA; Key Laboratory of Animal Genetics, Breeding and Reproduction of Shaanxi Province, College of Animal Science and Technology, Northwest A&F University, Yangling Shaanxi 712100, China; State Key Laboratory of Crop Stress Resistance and High-Efficiency Production, College of Horticulture, Northwest A&F University, Yangling Shaanxi 712100, China; Key Laboratory of Horticultural Plant Biology and Germplasm Innovation in Northwest China, Ministry of Agriculture and Rural Affairs, Yangling Shaanxi 712100, China

## Abstract

Grapevine powdery mildew (GPM), caused by *Erysiphe necator*, poses a significant threat to all green grapevine tissues, leading to substantial economic losses in viticulture. Traditional grapevine cultivars derived from *Vitis vinifera* are highly susceptible to GPM, whereas the wild Chinese accession Baishui-40 (BS-40) of *V. piasezkii* var. *pagnucii* exhibits robust resistance. To illuminate the genetic basis of resistance, we sequenced and assembled the chromosome-level genome of ‘BS-40’, achieving a total mapped length of 578.6 Mb distributed across nineteen chromosomes. A comprehensive annotation identified 897 nucleotide-binding leucine-rich repeat (NLR) genes in the ‘BS-40’ genome, which exhibited high sequence similarity across *Vitis* genomes. 284 of these NLR genes were differentially expressed upon GPM infection. A hybrid population of ‘BS-40’ and *V. vinifera* was constructed and 195 progenies were whole-genome re-sequenced. A new GPM-resistant locus, designated *Ren17*, located within the 0.74–1.23 Mb region on chromosome 1 was identified using genome-wide association study, population selection, and QTL analysis. Recombinant events indicated that an NLR gene cluster between 1 045 489 and 1 089 719 bp on chromosome 1 is possibly the key contributor to GPM resistance in ‘BS-40’. Based on an SNP within this region, a dCAPS marker was developed that can predict the GPM resistance in ‘BS-40’-derived materials with 99.4% accuracy in the progenies of ‘BS-40’ and *V. vinifera*. This chromosome-level genome assembly of *V. piasezkii* var. *pagnucii* provides a valuable resource not only for grapevine evolution, genetic analysis, and pan-genome studies but also a new locus *Ren17* as a promising target for GPM-resistant breeding in grapevine.

## Introduction

Grapevine is one of the most economically and culturally important horticultural crops worldwide, embodying the cultural identity of major Eurasian civilizations through its roles as a food source and winemaking ingredient [1]. However, cultivated grapevines, predominantly derived from *Vitis vinifera*, commonly are highly susceptible to different pathogens, necessitating substantial investments in disease prevention and control measures [2]. Among these challenges, grapevine powdery mildew (GPM), caused by the obligate biotrophic ascomycete fungus *Erysiphe necator*, could infect all green parts of grapevine and form white velvety mycelia on infected parts, causing 10%–45% yield losses to viticultural production and decrease of the wine quality, resulting in the heavy reliance on fungicides [3–6]. GPM management accounts for 74% of the total active ingredients applied to grapevines and up to 90% of the environmental impact of grape production in the western United States [7]. Breeding grapevine cultivars with inherent resistance to GPM offers a more sustainable and environmentally friendly solution to mitigating the losses caused by this disease. As a center of diversity of *E. necator*, eastern North America harbors species such as *V. rotundifolia*, *V. rupestris*, *V. riparia*, and *V. aestivalis* that have evolved resistance to GPM through host–pathogen co-evolution. Similarly, certain wild Chinese *Vitis* species, including *V. romanetii*, *V. piasezkii*, and *V. amurensis*, also exhibit qualitative resistance to GPM, highlighting their potential as valuable genetic resources for GPM resistance studies and breeding programs [8].

To explore the genetic evidence of GPM resistance in grapevine, a total of 15 GPM-resistant loci have been identified to date. The first identified locus, *Run1*, located on chromosome 12 of *V. rotundifolia* was found to trigger programmed cell death after the haustorium formation and was bred into *V. vinifera* to improve GPM resistance [9, 10]. With the *V. rotundifolia* hybrid background, quantitative loci *Run2.1*, *Run2.2* [11], and *Ren5* [12] have been identified. Recently, another locus *Ren11* identified on chromosome 15 from North America *V. aestivalis* was found to be effective in defending against the infection of *E. necator* in most vineyard environments [13]. Several GPM-resistant loci have also been identified in grapevine species with complex hybrid backgrounds, including *Ren2*, *Ren3*, *Ren9*, *Ren8*, and *Ren10* [14–17]. In addition, the GPM-resistant loci in wild Chinese species were mapped in *V. romanetii* (*Ren4*) [11], *V. piasezkii* (*Ren6* & *Ren7*) [4], and *V. amurensis* (*Ren12*) [8]. Among these GPM-resistant loci, *Run1*, *Ren4*, *Ren5, Ren6*, *Ren11*, and *Ren12* have been shown to act quickly and qualitatively reduce the formation of secondary hyphae on leaves that confer strong resistance to GPM [4, 8, 9, 12, 13, 18].

Traditional QTL analysis based on various DNA markers has emerged as a routine way for genetic mapping of interested traits. Nearly all the identified GPM-resistant loci were mapped using QTL except the earliest *Run1* and *Ren1* [4, 8, 10–17, 19]. Nevertheless, the variety and abundance of DNA markers have limited the QTL for fine mapping, leading to the precise identification of responsible genes for GPM resistance only in *Run1* locus to date [20]. With the rapid decline in sequencing costs, genome-wide DNA markers methodologies have been harnessed to explore candidate loci associated with grape disease resistance. For instance, based on hybrid populations, novel QTLs for the resistance to grapevine white rot [21] and downy mildew [22] were identified based on the genotyping by whole-genome resequencing. Even a new haplotype of known *Ren1* was found through genotyping by sequencing [23]. Beyond QTL analysis, the genome-wide association study (GWAS) has emerged as a prevalent approach for pinpointing interested loci, leveraging advancements in sequencing technology. With natural populations, candidate loci of grape berry-related traits were identified by GWAS and were consistent with previous QTL analysis [24]. Grapevine downy mildew [25] and ripe rot [26] resistance loci were also identified using GWAS in abundant genotypes. Interestingly, GWAS and QTL analysis could be applied simultaneously and support each other in hybrid populations, which helped the identification of development-related loci in peach [27] and novel downy mildew resistance locus in grapevine [28].

A reference genome is essential for biological and genetic studies. The first reference genome of grapevine was published in 2007 (8X), which used the highly homozygous material *V. vinifera* cv. Pinot Noir PN40024 derived from selfing of cv. ‘Helfensteiner’ [29]. Subsequently, updated versions of PN40024 genome were released in 2017 (12X.v2) and 2023 (PN40024.v4 & PN_T2T) [30–32]. Due to the high homozygosity, the PN40024 genome still stands as the reference for the grapevine community. More reference genomes are essential to satisfy the demands to study the function of other intra- and inter-species genotypes. Advancements in sequencing technologies and refinement of assembly methodologies have sparked a proliferation of genome assemblies for diverse grapevine cultivars, including ‘Cabernet Sauvignon’ [33], ‘Chardonnay’ [34], and ‘Merlot’ [35], among others. Furthermore, several genomes of wild *Vitis* species were assembled because of their specific outstanding traits, including *V. aestivalis* (resistant to major fungal disease and tolerant to freezing and fluctuating temperature) [36], *V. riparia* [37] and *V. rupestris* [38], as they both possess high abiotic and biotic stress tolerance and are globally employed in rootstock and scion breeding, *V. rotundifolia* (disease resistance and nutritional richness) [39], and *V. amurensis* (the most cold tolerant *Vitis* species) [40, 41]. The biography of *Vitis* genomics was reviewed comprehensively [42] and the pangenome analysis were performed recently [43, 44], which expanded the resources available for grapevine trait genetics and genomic breeding.

In this study, we sequenced and assembled a genome of Chinese wild grapevine accession Baishui-40 (hereafter abbreviated as ‘BS-40’) of *V. piasezkii* var. *pagnucii*. And a hybrid population was generated by crossing ‘BS-40’ (resistant to GPM) with *V. vinifera* cv. Pearl of Csaba (susceptible to GPM). Based on this hybrid population, a new GPM-resistant locus designated *Ren17* was identified, and candidate genes responsible for GPM resistance were fine-mapped. The genome of *V. piasezkii* var. *pagnucii* and the newly discovered GPM resistance locus will provide new valuable resources for molecular functional analysis and resistance breeding research in grapevine.

## Results

### Genome sequencing, assembly, and annotation of ‘BS-40’

**Figure 1:**
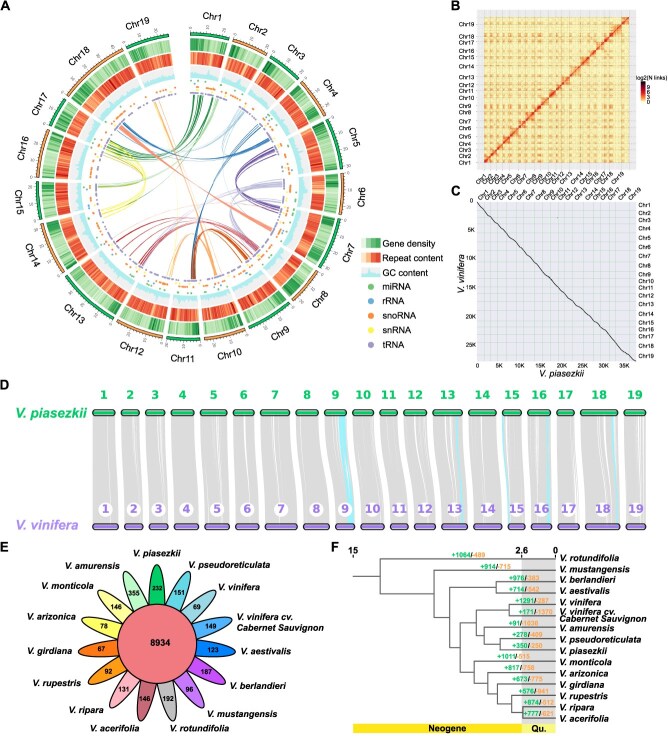
The genomic features of genome assembly of *Vitis piasezkii* var. *pagnucii* ‘BS-40’ and its evolutionary analysis. (A) A circos plot shows gene density, repeat content, GC content, miRNA, rRNA, snoRNA, snRNA, and tRNA on nineteen chromosomes of ‘BS-40’. The inner lines show the syntenic blocks in the ‘BS-40’ genome. (B) Hi-C interactions among nineteen chromosomes. The dark red line on the diagonal indicates the strong interactions. (C) Collinearity analysis and (D) synteny analysis between ‘BS-40’ and *V. vinifera* cv. PN40024. The inverted regions were marked with blue ribbons. (E) Venn diagram shows the shared and unique gene families among ‘BS-40’ and other 14 *Vitis* genomes. (F) The phylogenetic relationship and divergence times (Mya, black numbers) based on all single-copy gene families shared by 15 *Vitis* genomes. The colored numbers represent these expansion (green) and contraction (orange) gene families.

‘BS-40’ is a dioecious male accession of wild Chinese grapevine *V. piasezkii* var. *pagnucii* (Fig. S1), which was identified with high resistance to GPM [45]. The genome survey showed that it possesses high heterozygous levels (Fig. S2). To perform de novo assembly of ‘BS-40’ genome, the Oxford Nanopore, Illumina Hiseq, and high-throughput chromosome conformation capture (Hi-C) platforms were combined. We generated 148-fold-coverage Nanopore data (84.83 Gb), 80-fold-coverage Illumina data (45.69 Gb), and 89-fold-coverage Hi-C data (51 Gb). The nanopore reads were corrected and assembled, finally generated an assembly comprising 509 contigs with an N50 of 2.08 Mb (Table 1). These contigs were then corrected and polished with Illumina Hiseq sequence. 567 Mb (98.12%) Hi-C paired-end reads were located on 19 pseudo-chromosomes. Combining with Hi-C data, a final genome of 578.64 Mb was assembled, which could be scaffolded on 19 chromosomes (Fig. 1A and B). These chromosomes ranged from 18.6 Mb to 44.3 Mb and were named according to the *V. vinifera* PN_T2T (https://www.ncbi.nlm.nih.gov/datasets/genome/GCF_030704535.1) (Table S1).

**Table 1:** Comparison of genome assembly global statistics between ‘BS-40’ and other Vitis species genomes.

Assembly feature	BS-40 (*V. piasezkii*)	*V. amurensis*	*V. aestivalis*	*V cinerea*	*V. labrusca*	*V. ripara*	*V. rupestris*	PN40024_12X (*V. vinifera*)	PN40024_T2T (*V. vinifera*)
Genome size (Mb)	578.64	522.28	432.8	497.9	570.6	500.1	607.1	485.3	494.9
Contig N50 (Mb)	2.08	2.5	0.000772	0.04	0.26	0.74	0.03	0.1	26.9
Number of contigs	509	615	756 125	39 902	39 288	1160	45 770	14 632	19
Scaffold N50 (Mb)	26.8	26.5		0.27	0.46	23.9	0.37	3.4	26.9
Number of scaffolds	333			26 134	9752	174	29 931	2059	19
GC content (%)	34.56	34.5	34	34.5	34.5	34.5	35	34.5	35
Number of genes	41 251	27 635				30 745		29 956	29 591
Complete BUSCOs (%)	94.9	97.5				98.8		99.3	98.8

The integrality of the assembled *V. piasezkii* var. *pagnucii* genome was assessed by CEGMA v2.5, BUSCO v2.0, and Illumina Hiseq data. 93.89% CEGs (Core Eukaryotic Genes) of the CEGMA database were blasted in the ‘BS-40’ genome. 1367 (94.93%) complete BUSCOs were identified (Table 1) and 1312 (91.11%) were single-copy BUSCO genes. In addition, Illumina Hiseq short reads were mapped to the assembled genome with bwa, and 98.59% of reads were mapped. These results indicated high accuracy and integrality of the chromosome-level ‘BS-40’ genome.

About, 351 Mb of repetitive sequences was annotated, accounting for 60.66% of the assembled ‘BS-40’ genome (Fig. 1A; Table S2). A total of 41 251 protein-coding genes were identified based on the *ab initio* prediction, homologous search, and RNA-seq data, with an average gene length of 4786.8 bp, coding sequence length of 1152.4 bp, and intron length of 3408.3 bp (Table S3). These predicted protein-coding genes were annotated using NR, KOG, GO, KEGG, and TrEMBL databases using BLAST v2.2.31 (e-value 1e^−5^), achieving a 98.00% functional annotation rate (Table S4). In addition, non-coding RNA, including 147 micro-RNAs, 149 ribosomal RNAs, 418 snoRNAs, 94 snRNAs, and 507 transfer RNAs were annotated (Table S5).

### Evolutionary and gene family analysis of the BS-40 genome

The syntenic analysis of ‘BS-40’ and other 14 *Vitis* genomes, including 2 other Chinese species (*V. amurensis* and *V. pseudoreticulata*), 2 cultivars of European species (*V. vinifera* cv. PN40024 and *V. vinifera* cv. Cabernet Sauvignon), and 10 American species (*V. acerifolia*, *V. aestivalis*, *V. arizonica*, *V. berlandieri*, *V. girdiana*, *V. monticola*, *V. mustangensis*, *V. ripara*, *V. rotundifolia*, and *V. rupestris*), was performed to classify the differences between these genomes. High collinearity was observed between ‘BS-40’ and other *Vitis* genomes (Fig. S3). Specifically, a total of 19 382 gene pairs were identified between ‘BS-40’ and *V. vinifera* cv. PN40024, demonstrating high collinearity in both pairwise synteny and the more succinct macrosynteny analysis (Fig. 1C and D).

To further generate the characteristics and evolution of the ‘BS-40’, a comparative genomic investigation of 15 *Vitis* genomes was conducted. 616 029 (97.5%) genes were assigned in 37 938 gene families, of which 232 gene families were unique in ‘BS-40’ (Fig. 1E). A total of 636 unique genes were identified in these unique gene families (Table S6), and these unique genes were enriched in GO and KEGG pathways. The RNA–DNA hybrid ribonuclease activity and primary metabolic process were significantly enriched in GO. The endocytosis, spliceosome, and phagosome pathways were enriched significantly in KEGG (Fig. S4).

A high-confidence phylogenetic tree was generated using protein sequences from 1170 single-copy gene families between ‘BS-40’ and the fourteen *Vitis* genomes above mentioned while *V. rotundifolia* was selected as an outgroup (Fig. 1F). ‘BS-40’ and other Chinese grapevine, *V. amurensis* and *V. pseudoreticulata*, were clustered in one monophyletic group with European cultivars *V. vinifera* cv. PN40024 and *V. vinifera* cv. Cabernet Sauvignon. There were 350 expanded and 250 contracted gene families in the ‘BS-40’ genome (Fig. 1F). The expanded gene families, including 3201 genes, were enriched mainly in the plant-pathogen interaction, alpha-Linolenic acid metabolism, and glutathione metabolism pathways (Fig. S5, Table S7). About, 374 genes in the contracted families were predicted to function in the Circadian rhythm—plant, flavonoid biosynthesis, and plant–pathogen interaction pathways (Table S8).

### The classification, distribution, and expression of NLR genes of ‘BS-40’

**Figure 2:**
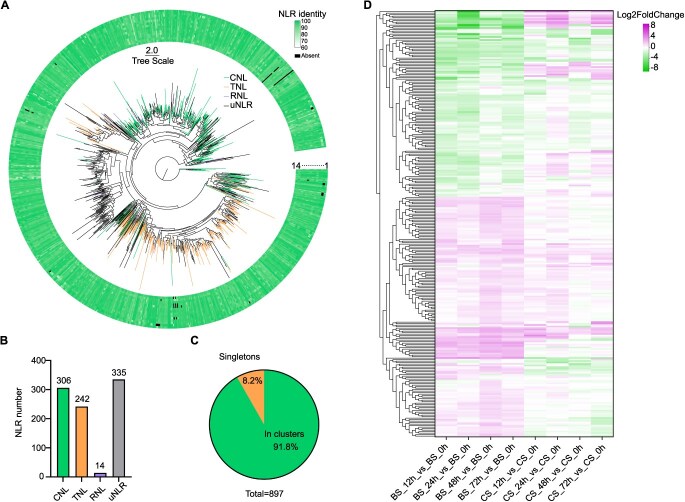
The maximum-like tree and expression of NLRs in the ‘BS-40’ genome. (A) The NLRs of ‘BS-40’ were classified into CNLs (green), TNLs (orange), RNLs (purple), and uncharacterized NLRs (black). All 897 NLRs were used to construct a maximum-like tree by IQ-TREE. The Present/Absent polymorphism of these NLRs from 14 other *Vitis* genomes are shown by the heatmap (white to green, black blocks represent the absent NLRs). The species order from outside to inside (1–14) represents *V. amurensis*, *V. pseudoreticulata*, *V. vinifera* cv. PN40024, *V. vinifera* cv. Cabernet Sauvignon, *V. acerifolia*, *V. aestivalis*, *V. arizonica*, *V. berlandieri*, *V. girdiana*, *V. monticola*, *V. mustangensis*, *V. ripara*, *V. rotundifolia*, *and V. rupestris,* respectively. The tree scale bar represents the number of amino acid substitutions per site. (B) The number of CNLs, TNLs, RNLs, and uncharacterized NLRs in the ‘BS-40’ genome. (C) The proportion of NLR singletons and NLRs in clusters. (D) The expression of NLRs in ‘BS-40’ and *V. vinifera* cv. Cabernet Sauvignon (CS) at 12, 24, 48, and 72 hours postinoculation with powdery mildew.

Resistance (*R*) genes play key roles in plants' response to biotic stress. One common characteristic of these *R* genes is that most of them possess NB-ARC (nucleotide-binding domain shared by APAF-1, various plant *R* proteins and CED-4) and LRR (leucine-rich repeat) domains, which results in coding NLR proteins. The distribution of NLR genes in the ‘BS-40’ genome was identified according to the genome annotation and NLR-annotator prediction results. As listed in Table S9, there are 897 genes annotated as NLR genes on the 19 chromosomes, which could be translated to complete or partly NLR proteins. To understand the diversity of these NLR genes, a phylogenetic tree was generated using the amino acid sequences (Fig. 2A). The presence/absence polymorphisms of these genes among other 14 *Vitis* genomes were compared and high similarity identity of these genes was observed (Fig. 2A; Table S10).

The N-terminal domains of NLR proteins could be classified into three types: coiled-coil (CC), Toll/interleukin-1 receptor/resistance protein (TIR), and RPW8-like coiled-coil domain (RPW8), resulting in three types of NLRs: CNLs, TNLs, and RNLs. In ‘BS-40’, 306 CNLs, 242 TNLs, and 14 RNLs were identified (Figs 2B and S6). The others were uncharacteristic NLR genes (uNLR) as their N-terminal lack specific domains. Besides, 91.8% of these NLR genes were in clusters and the rest were singletons (Figs 2C and S6). In terms of distribution, these NLR genes are mainly located at chromosome 9 (12.7%), 12 (10.8%), 13 (16.8%), and 18 (26.4%) (Fig. S6). And NLR gene clusters were also observed at chromosome 1, 5, 7, 14, 15, and 19.

To identify the expression patterns of these NLR genes under biotic stress, the young leaves of ‘BS-40’ were inoculated with an isolate of GPM pathogen *Erysiphe necator*, *En.* NAFU1. The leaves at 0, 12, 24, 48, and 72 h postinoculation (hpi) were collected to perform transcriptome analysis. A susceptible material *V. vinifera* cv. Cabernet Sauvignon (CS) was used as control. The results showed that 284 (31.7%) NLR genes of ‘BS-40’ were differentially expressed (*P*adj <0.05 & |Log2FoldChange| > 1) following the infection of *En.* NAFU1. These significantly differentially expressed NLR genes between ‘BS-40’ and ‘CS’ were annotated mainly as disease resistance protein *RPM1-*like, *RPP-*like, *RPS2-*like, *RGA4*, *RPW8-*like, and *TMV* resistance protein (Fig. 2D; Table S11).

The NLR genes of ‘BS-40’ were compared with the unique and expanded genes to explore their relationship. The results showed that eight NLR genes of ‘BS-40’ were unique, and 297 NLR genes of ‘BS-40’ were expanded (Fig. S7, Tables S12 and S13). These eight unique NLR genes were mainly annotated as encoding At1g12280 and RPP13-like protein (Table S12). While these expanded NLR genes were annotated as disease resistance protein RPM1-like, RPP13-like, RGA4, and TMV resistance protein (Table S13). All expanded genes in the ‘BS-40’ genome were not unique. In other words, these unique genes of ‘BS-40’ were not related to gene expansion. These results suggested that abundant NLR genes of ‘BS-40’ originated from gene expansion events, which may have important functions in response to biotic stress such as GPM.

### A new locus *Ren17* is highly associated with grapevine powdery mildew resistance in ‘BS-40’

**Figure 3:**
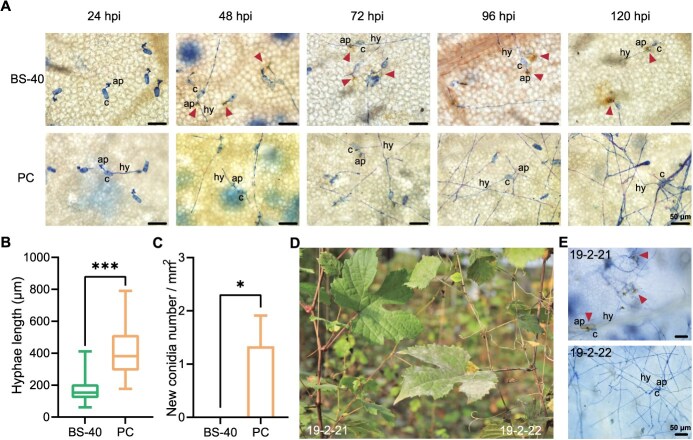
The resistance level to powdery mildew of *V. piasezkii* var. *pagnucii* ‘BS-40’, *V. vinifera* cv. Pearl of Csaba, and their crossing progenies. (A) Trypan blue stained fungal structure and Diaminobenzidine (DAB) stained dead epidermal cells on ‘BS-40’ and Pearl of Csaba (PC) leaves inoculated with powdery mildew *En.* NAFU1. The conidium (c), appressoria (ap), and hyphae (hy) of *En.* NAFU1 were stained blue. The red triangle pointed to the H_2_O_2_ accumulation in the cell death progress. (B) The hyphae length of *En.* NAFU1 at 72 hpi on ‘BS-40’ and ‘PC’. *n* = 30, *P* < 0.001. (C) The new conidium number of *En.* NAFU1 at 120 hpi on ‘BS-40’ and ‘PC’. *n* = 3, *P* < 0.05. (D) The leaves of crossing progenies from ‘PC’ × ‘BS-40’ at 14 days post inoculation with *En.* NAFU1. (E) The fungal structure and cell death on detached leaves of crossing progenies at 3 days post inoculation with *En.* NAFU1*.* bar = 50 μm.

Previous studies have shown that ‘BS-40’ possesses high resistance to GPM [45]. The GPM resistance of ‘BS-40’ was validated again in this research by inoculation with the pathogen *En.* NAFU1. As shown in Fig. 3A, the hyphae growth of *En.* NAFU1 stained with the trypan blue and diaminobenzidine was inhibited significantly and program cell death caused by the accumulation of H_2_O_2_ was observed on ‘BS-40’ leaves, which showed a high resistance level to GPM.

To further identify the genetic evidence of GPM resistance, a hybrid population was conducted by crossing ‘BS-40’ with *V. vinifera* cv. Pearl of Csaba (PC), which is susceptible to GPM (Figs 3A–C and S8A). A total of 218 F_1_ individuals were generated, of which 106 were resistant to GPM while 106 were susceptible, and 6 individuals were undetected because of unhealthy growth conditions (Figs 3D, E and S8). The resistance levels to GPM of these individuals were observed in field and laboratory four times from 2020 to 2022 (Table S14). With an observed 1:1 Mendelian ratio, the resistance to GPM in ‘BS-40’ was speculated as a monogenic dominant genetic control (χ^2^ = 0; *P* > 0.05).

**Figure 4:**
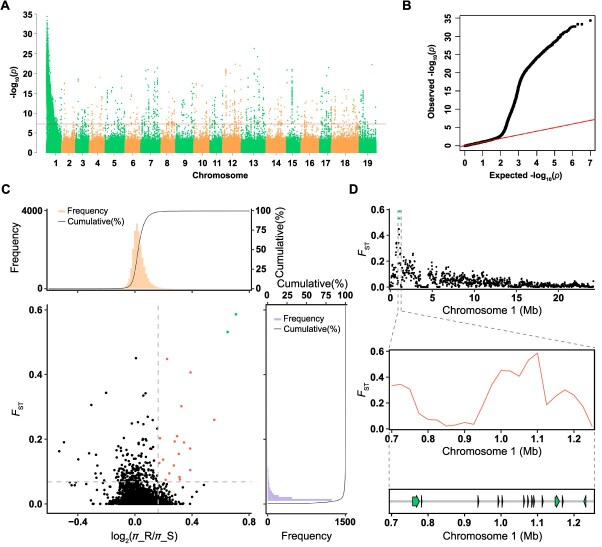
A locus on chr1 associated with powdery mildew (PM) resistance was identified using Genome-wide association study (GWAS) and population selection analysis. (A) Manhattan plot and (B) Q-Q plot of the GWAS analysis for PM resistance in the crossing progenies from ‘PC’ × ‘BS-40’. A locus highly associated with PM resistance was identified as *Ren17* on chr1. The red horizontal line in Manhattan plot depicts the significance threshold (−log10 (*P*) = 7.5). (C) Distribution of population differentiation (*F*_ST_) and *π* ratios (log2(*π*_R/*π*_S)) between resistant (R) and susceptible (S) progenies. Dots in the upper right (marked in red) are genomic regions with high population differentiation and high nucleotide diversity, with both values above the 99% threshold. The top two selected regions are marked in green. (D) *F*_ST_ values of SNPs adjacent to the top two selected regions. The green arrows with black frame are the resistance genes distributed in *Ren17*.

To further explore the genetic basis, 195 F_1_ individuals and the female parent ‘PC’ were genome re-sequenced and mapped to the ‘BS-40’ reference genome. 4 815 045 filtered single nucleotide polymorphisms (SNPs) were generated for further analysis (Fig. S9A). Principal component analysis (PCA) showed that most of the progenies were different from their female parent ‘PC’, except two individuals 19-2-31 and 19-2-75, which were regarded as the female parent genotype in the next analysis (Fig. S9B). Variants from 171 progenies (well grown and have stable resistance levels to PM, in which 84 progenies were resistant and 87 were susceptible, Table S14) were selected to carry out the genome-wide association study (GWAS) with female parent ‘PC’. The results showed that a major locus on chromosome 1 (0.74−1.23 Mb, −log10 (*P*) > 33) was associated with the resistance to GPM most significantly (Figs 4A, B and S10; Table S15), hereafter named as *Ren17* (*Resistance to Erysiphe necator 17*) according to the naming rules of VIVC (*Vitis* International Variety Catalogue) database.

According to the genome annotation results, a total of 41 genes were located in the *Ren17* locus (Table S16). Among these genes, 13 were annotated as disease resistance protein *RPM1*, *RPM1-*like and *RGA*. These 13 NLR genes may function in the signal transduction mechanisms based on the KOG class annotation. And 8 of them were categorized as defense response in GO annotation. Notably, these 13 NLR genes were clustered in chromosome 1 (Fig. S6). In addition, some genes related to nucleic acid binding, calcium ion binding, RNA splicing, and protein peptidyl-prolyl isomerization are also located in the *Ren17* locus.

Population selection and QTL analysis were carried out to verify the GWAS results. For the population selection analysis, the genome regions, with extreme allele frequency divergence and the greatest differences in nucleotide diversity between the resistant (R) and susceptible (S) populations, were identified as candidate divergent regions (CDRs). Regions under selection usually show high population differentiation (*F*_ST_) and low nucleotide diversity (*π*) [46]. Twenty regions in this study observed increased differentiation levels between R and S populations and significant difference in nucleotide diversity (high nucleotide diversity in resistant individuals and low nucleotide diversity in susceptible individuals) (*F*_ST_ > 0.07 & log_2_(*π*_R/*π*_S) > 0.17, top 1% threshold) (Fig. 4C; Table S17). The top 2 among these regions possessed high selective signals (*F*_ST_ > 0.53 & log_2_(*π*_R/*π*_S) > 0.64) were ranged from 1 075 001 to 1 125 000 bp on chromosome 1 (Fig. 4D; Table S17). In the QTL analysis based on the genotype and GPM phenotype, the most significant signal on chromosome 1 was detected at the SNP chr1_856772 (LOD = 135, *P* < 0.01) (Fig. S11). These segments identified by population selection and QTL analysis were also located in the *Ren17* locus supporting the association between *Ren17* and GPM resistance.

The gene-level synteny analysis of the *Ren17* locus was carried out to reveal the difference of this locus between the wild Chinese grapevine ‘BS-40’ and *V. vinifera* cv. PN40024. The results showed that these 13 NLR genes in ‘BS-40’ have high synteny with PN40024 (For easy to refer, these 13 NLR genes in the *Ren17* locus were named from R1 to R13, see Table S16.) Many-to-one synteny relationships (e.g. R2 and R8 to LOC100244008) were observed in addition to one-to-one synteny (e.g. R9 to LOC109123391) (Fig. S12).

### *RPM1/RPM1-like* genes in the *Ren17* locus may be crucial to the GPM-resistance in ‘BS-40’

**Figure 5:**
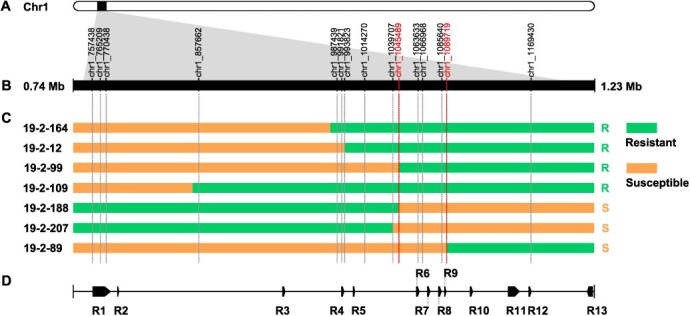
Recombinants analysis of the *Ren17* locus. (A) The physical position of the *Ren17* locus on chromosome 1. (B) The SNPs used for recombinants analysis. (C) Genotype and phenotype of the crossing progenies. The numbers on the left side are the IDs of the crossing progenies. Green bars represent the resistant haplotype from the heterozygous genotype of ‘BS-40’. Orange bars represent the susceptible haplotype from ‘BS-40’, which causes the homozygous genotype same as ‘PC’. R (resistant) and S (susceptible) on the right side are the resistance levels to powdery mildew. (D) Resistance genes cluster in the *Ren17* locus.

To identify which genes may be responsible for the GPM-resistance in ‘BS-40’ more precisely, 15 SNPs highly associated with GPM-resistance in the *Ren17* locus were selected to explore SNP markers (Fig. 5B; Table S18). Recombinant events were screened in the *Ren17* locus in the F_1_ individuals. Results of seven representative individuals showed that the genotypes were highly conserved between chr1_1 045 489 and chr1_1 089 719 and clearly distinguished the resistant and susceptible individuals (Fig. 5C). There are four NLR genes, including R6, R7, R8, and R9, located in this region, which were considered as candidate genes that contribute to the GPM-resistance in ‘BS-40’ (Fig. 5D). Furthermore, the *F*_ST_ of locus *Ren17* also showed a high difference between resistant and susceptible individuals around the R6 to R9 region (Fig. 4D).

The gene structure and coding sequence domains showed that all of these 13 NLR genes located in *Ren17* locus coding complete or part of CNL proteins (Fig. S13). R1-R12 possess high phylogeny relationships with *RPM1* or *RPM1-like* genes in *V. vinifera* cv. PN40024 but differ from the *RPM1* in other plants, while R13 has high similarity with *RGA1* (Fig. S14; Table S19). The transcriptome results showed that three (R3, R8, R10, and R13) of these 13 NLR genes were differentially expressed significantly (*P*adj <0.05 and |log2FoldChange| >1) in ‘BS-40’ during the powdery mildew infection. R3, R8, and R10 were down-regulated firstly post GPM infection and then the expression level of R3 and R8 increased slightly at 72 hpi, and R10 increased from 24 hpi. The expression of R13 was up-regulated in ‘BS-40’ and down-regulated in *V. vinifera* cv. Cabernet Sauvignon (Table S11).

### dCAPS marker developed in the *Ren17* locus

**Figure 6:**
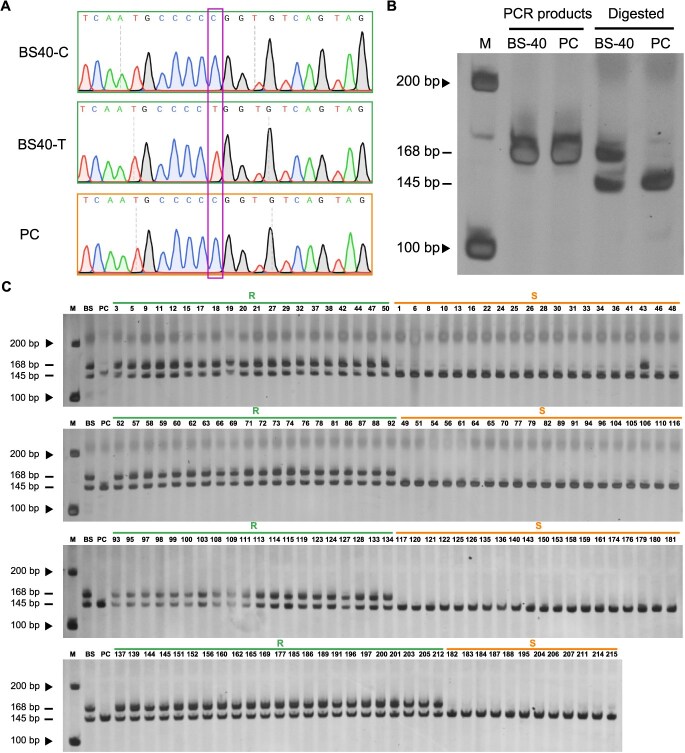
A dCAPS marker based on SNP chr1_1066968 was developed to detect the GPM resistance of ‘BS-40’-derived materials. (A) The sequenced chromatogram of the genotypes at chr1_1066968 in ‘BS-40’ and ‘PC’. The purple box indicated the polymorphism of chr1_1066968. (B) The PCR products and digested fragments by restriction endonuclease *Sma* I of nucleotides adjacent to the chr1_1066968 in ‘BS-40’ and ‘PC’. (C) The digestion results of chr1_1066968 adjacent nucleotides in ‘BS-40’, ‘PC’, and their 154 progenies. ‘R’ represents these progenies are resistant to GPM, while ‘S’ represents these progenies are susceptible. The numbers are the IDs of 154 progenies.

To detect the presence of *Ren17* locus in ‘BS-40’ and its derived materials faster, easier, and cost-lower, molecular markers were developed based on these 15 SNPs associated with GPM above mentioned (Fig. 5). As no common restriction enzyme recognition sites were found around these SNPs, the derived-cleaved amplified polymorphic sequence (dCAPS) was developed by dCAPS Finder 2.0 to genotype these SNPs. One SNP, chr1_1066968 (T/C), was highly linked to GPM (Fig. 5) and observed in polymorphic bands via enzyme digestion (Fig. 6A and B). The five nucleotides before/behind the SNP were GCCCC (T/C) GGTGT, when the SNP was ‘C’ and the third nucleotide behind the mutant site was mismatched changing ‘T’ to ‘G’ by the dCAPS primer, *Sma* I recognition site (CCCGGG) was generated adjacent to the SNP; when the SNP was ‘T’, the recognition site was absent. The primer pair could amplify a 168-bp fragment, which with the recognition site could be digested into 145 and 23-bp fragments by *Sma* I.

The sequencing results showed that there are ‘T’ and ‘C’ genotypes in ‘BS-40’, while ‘PC’ only possesses ‘C’ (Fig. 6A). Hence, there are two bands when the amplicons of ‘BS-40’ digested by *Sma* I because the ‘C’ genotype was digested while the ‘T’ genotype could not. Meanwhile, only a 145-bp band was observed after the amplicons of ‘PC’ were digested (Fig. 6B). 154 F_1_ progenies of ‘BS-40’ and ‘PC’ were also detected by this dCAPS marker. The 168-bp fragments could be amplified from all of these progenies (Fig. S15) and 99.4% (153/154) of the digestion results were consistent with their resistance to GPM (Fig. 6C). According to these results, this dCAPS marker could be used to detect the GPM resistance level of ‘BS-40’-derived materials at the seedling stage and accelerate the GPM-resistance breeding progress of grapevine.

## Discussion

A high-quality reference genome is essential for biological research, genetic studies, and breeding programs. Grapevine is one of the most economically and culturally important horticultural crops, with different versions of *Vitis* species genomes have been assembled and released since the first publication of the PN40024 genome in 2007 [29]. Despite the genomes of European grapevine *V. vinifera* were assembled and annotated more and more completely, challenges persist in searching its genetic evidence for disease resistance, primarily due to the absence of selective pressures imposed by diseases like GPM [8]. Thus, several genomes of *Vitis* species renowned for their robust environmental adaptability have been assembled, aiming to explore the genetic resources for both biotic and abiotic resistance [36–41]. Here, we sequenced and assembled the genome of *V. piasezkii* var. *pagnucii*, a wild grapevine originated from China that exhibits remarkable resistance to GPM [4, 45], with Nanopore long reads technology in 2019. Even though the quality of our *V. piasezkii* var. *pagnucii* genome is lower than the most recently published PN_T2T (telomere-to-telomere level genome of *V. vinifera*), it still possesses much higher Contig N50 (2.08 Mb) than the previous version of *V. vinifera* (PN40024_12X.v0, 0.1 Mb) and most of other initial reference genomes of grapevine such as *V. aestivalis* (0.000772 Mb), *V. ripara* (0.074 Mb), *V. rupestris* (0.03 Mb), and close to *V. amurensis* (2.5 Mb) (Table 1). With generated 148-fold-coverage Nanopore data (84.83 Gb), 80-fold-coverage Illumina data (45.69 Gb), and 89-fold-coverage Hi-C data (51 Gb), it could be used as a high-quality initial reference genome of *V. piasezkii* var. *pagnucii*. Recently, a haplotype genome of *V. piasezkii* with a higher Contig N50 (26.2 Mb) was released based on PacBio HiFi and Hi-C data, suggesting that the development of sequencing technology indeed improves the genome quality [47]. As the high GPM resistance of *V. piasezkii* var. *pagnucii* possesses, our genome could be used as an important reference for grapevine resistance breeding along with the haplotype genome. Given that limitations exist in haploid genomes, assembling two haplotypes of *V. piasezkii* var. *pagnucii* will provide more precise information in the future.

Contrary to the general assumption that GPM originated from North America, wild Chinese grapevine species exhibit resistance to GPM, suggesting distinct resistance mechanisms that may have evolved independently [4]. However, high synteny was observed between *V. piasezkii* var. *pagnucii* and *V. vinifera* cv. PN40024 (Fig. 1). Notably, the genome size is the most obvious difference between these two genomes. The genome of ‘BS-40’ has a bigger size than that of PN40024 (Table 1), potentially attributed to the high percentages of repetitive sequences and more annotated genes in the ‘BS-40’ genome, which needs to further validation based on haplotypes genome. The comparative genomic analysis with 14 *Vitis* genomes revealed the specificity and evolutionary dynamics of ‘BS-40’. The unique gene of ‘BS-40’ enriched in the plant-pathogen interaction pathway mainly annotated as *RPS2* (Table S6). In *Arabidopsis thaliana*, *RPS2* was reported to confer resistance against *Pseudomonas syringae* bacteria by cooperating with *RPM1* [48, 49]. The expanded gene families identified in ‘BS-40’ were primarily enriched in the plant-pathogen interaction, alpha-linolenic acid metabolism, and glutathione metabolism pathways (Fig. S5, Table S7). The expanded genes enriched in the plant–pathogen interaction pathway were annotated as disease resistance proteins RPM1 and RPS2, which confer resistance to pathogens in Arabidopsis [48, 49]. Interestingly, some differentially expressed genes in transcriptome analysis of ‘BS-40’ upon powdery mildew infection were also annotated as *RPM1* and *RPS2*. (Table S11). The alpha-linolenic acid was reported not only as a strong antioxidant, but also could act as a precursor to the synthesis of JA, which acts as a signaling molecule to stimulate the downstream stress response [50]. The glutathione metabolism pathway is fundamental to plant survival, notably for its pivotal role in regulating reactive oxygen species (ROS). Meanwhile, the biosynthesis and degradation of glutathione are regulated by biotic and abiotic stress [51]. These results suggest that these unique and repeated gene families in ‘BS-40’ may represent an evolutionary adaptation to diverse environmental stress, potentially playing crucial roles in enhancing resistance. It is worth to notice that different assembly approaches of these genomes in comparative genomic analysis may impact the evolution analysis results, and haplotype-scale comparison will help understand the differences more precisely.

Pathogens and plants continuously engage in genetic innovation and preservation of long-standing variations on both sides for infection or defense. To prevent the infection, plants armed pathogen-associated molecular patterns (PAMPs), which could trigger a cascade of cellular responses, ultimately preventing pathogen colonization, known as PAMP or pattern-triggered immunity (PTI). To overcome the PTI of hosts, pathogens often deliver effectors into plant cells to evade or suppress this immunity mechanism. Consequently, plants have evolved resistance (*R*) genes that specifically recognize pathogen effectors, eliciting a robust defense response called effector-triggered immunity (ETI) [52]. Given the pivotal role of *R* genes in ETI, genome-wide screening of these genes offers valuable insights into plant resistance to pathogens. In this study, abundant *R* genes that coded nucleotide-binding leucine-rich repeat receptors (NLRs) were annotated within the ‘BS-40’ genome (Figs 2A and S6), displaying an expansion compared to ancestors (Figs 1F and S7; Table S7). Similar to other wild progenitors, the NLRs in ‘BS-40’ (897) are more than reported *Vitis* cultivars [53]. Despite high similarity identities observed among NLRs of ‘BS-40’ and other *Vitis* genomes, alignment analysis revealed that multiple NLRs of ‘BS-40’ aligned to the same NLR from the other species. This finding underscores the greater abundance and repetitive nature of NLRs in ‘BS-40’ compared to other *Vitis* genomes (Fig. 2A; Table S10). Another significant characteristic of NLRs is their clustered genomic arrangement, which facilitates the generation of novel functional diversity through unequal crossovers and gene conversion [54]. Any deletion will lead to truncation or loss of a single-gene loci. In contrast, repeats in a cluster provide NLR genes with a broader and more flexible genetic basis for evolving new resistance specificities [55]. In this study, 91.8% (823) of ‘BS-40’ NLRs were found in clusters (Fig. 2C), which is more than other 17 reported *Vitis* species using a different definition of gene clusters [53], reflecting the evolution of ‘BS-40’ facing a complex natural environment.

GWAS has effectively pinpointed candidate genes that regulate crucial agronomic traits in grapes, including skin color, development periods, berry weight, flesh texture, and flavor [24]. These GWAS findings are consistent with QTL analyses conducted in both natural [24] and biparental populations [27]. In this study, GWAS was employed to explore the genetic locus responsible for GPM resistance based on the whole-genome resequencing data. As the genome survey showed (Fig. S2), ‘BS-40’ is a high heterozygous accession that could help generate substantial genotypic diversity in offspring. A locus, designated *Ren17*, was identified on chromosome 1 within the 0.74–1.23 Mb region, which displays a significant association with GPM resistance (Fig. 4A). Considering that interferential signals were observed on other chromosomes, population selection and QTL analysis were performed to support the GWAS findings. The population selection analysis highlighted genome regions with high levels of differentiation and significant nucleotide diversity [46]. Here, two significant regions that coincide with the *Ren17* locus were identified by separating the re-sequenced samples into resistant (R) and susceptible (S) populations to stimulate selection (Fig. 4C; Table S17). Consistently, the most significant QTL signal located in the *Ren17* locus as well (Fig. S11). These results approved that GWAS could be used as a meaningful tool to explore candidate locus in high heterozygous bi-parental populations and reinforced the hypothesis that the genes within *Ren17* may be responsible for GPM resistance in ‘BS-40’.

Previous research has identified two GPM-resistant loci, *Ren6* & *Ren7*, located on chromosome 9 and 19, respectively, in *V. piasezkii* [4]. However, no analogous signals were detected in ‘BS-40’ (Fig. S10), suggesting potential genetic divergence and differing resistance mechanisms between ‘BS-40’ (*V. piasezkii* var. *pagucii*, originating from Shaanxi province) and DVIT2027 (*V. piasezkii* Maxim., sourced from Hubei province), which deserves further verification. And these two varieties show a significant difference in morphology that the abaxial surface of the leaves in *Vitis piasezkii* var. *pagnucii* lacks pubescence. In addition, the gene-level synteny of the *Ren17* locus between ‘BS-40’ and *V. vinifera* cv. PN40024 indicated that these 13 NLRs within the *Ren17* locus have synteny with these genes of PN40024 (Fig. S12). The phenomenon of many-to-one (e.g. R2 and R8 to LOC100244008, or R4, R5, and R11 to LOC100256051) may be attributed to gene expansion events. The NLRs in *Ren17* locus were mainly annotated as *RPM1* or *RPM1-*like genes (Fig. S14). While *RPM1* is typically known for its ability to recognize bacterial effectors [56], it has also been implicated in response to fungal infection in grapevine and wheat [57, 58]. Transcription levels revealed that some of *RPM1* genes in *Ren17* responded to *E. necator* infection (Fig. 2D; Table S11). As the NLR genes could be constitutive, the function of these *RPM1* genes needs to be further verified in grapevine. Moreover, the pyramiding of additional resistance loci is necessary for effective GPM-resistant grapevine breeding programs, given these NLR genes generally confer race-specific resistance. And the dCAPS marker developed in this study could detect GPM resistance in ‘BS-40’ derived materials during the seedling stage, thereby accelerating the breeding of GPM-resistant grapevines.

In summary, this study provided a new genome resource of wild grapevine and novel genetic insights into GPM resistance. The cross population provided valuable germplasm resources for grapevine resistance breeding. The developed dCAPS marker can be used to accelerate the GPM resistance breeding progress of grapevines.

## Materials and methods

### Plant materials

The Chinese wild grapevine *V. piasezkii* var. *pagnucii* accession Baishui-40 (BS-40) was collected in Baishui County, Shaanxi, China, and kept with the *V. vinifera* cv. Pearl of Csaba (PC) in the germplasm resource repository of Northwest A&F University, Shaanxi, China. An F_1_ population of 218 individuals, generated from a cross between ‘PC’ (female parent, susceptible to powdery mildew) and ‘BS-40’ (male parent, resistant to powdery mildew) in 2019, was planted and maintained in a greenhouse of Northwest A&F University from 2020. All plant materials were cultured under normal conditions.

### Genome assembly

A genome survey of ‘BS-40’ has been carried out before the genome sequencing and assembly. Young leaves of ‘BS-40’ were collected to extract genomic DNA and construct the library following Illumina standard protocols. The library was sequenced by the Hiseq platform in paired-end 150 bp mode, and the clean reads were used to assess the genome size, GC density, and heterozygosity. The parameter *k* = 19 was used to show the k-mer distribution. The depth of the major peak represented the average k-mer depth, while the double depth of the average depth was considered as the repeat sequences, and the half depth of the average depth represented the heterozygous sequences. The genome size of ‘BS-40’ was estimated preliminarily with the formula: Genome size = total k-mer number/Average k-mer depth.

To guarantee the purity of the sample, tissue-cultured embryos of ‘BS-40’ were used to extract genomic DNA for genome assembly. The high-quality genomic DNA of BS-40 was extracted, purified, and integrity-detected following the standard protocols offered by Oxford Nanopore Technologies (ONT). The library was constructed by Biomarker Technologies (Beijing, China) and sequenced by the Nanopore platform. Clean data were generated by filtering the low-quality reads and removing adaptors. After being corrected by Canu (v1.8), SMARTdenovo was used to assemble the primary genome. Combining the genome survey sequences, the primary genome was corrected again with Pilon (v1.23) to improve the genome quality. CEGMA (v2.5), BUSCO (v2.0), and bwa (v0.7.17) were used to evaluate the integrity of the assembled genome.

### Hi-C library supports the genome assembly

To facilitate the genome assembly to the chromosome level, a Hi-C library was constructed with the tissue culture seedlings of ‘BS-40’. 2 g young leaves were collected and incubated in 2% formaldehyde solution with 35 mL pre-cold buffer (20 mM HEPES (pH 8), 250 mM sucrose, 1 mM MgCl_2_, 40% Glycerin, 0.25% Triton X-100, 0.1 mM PMSF, and 0.1% β-mercaptoethanol) at 25°C for 1.5 h. Add 2.5 ml 2 M glycine and shake gently for 5 min. The plant tissue was filtered with a nylon net, washed with ddH_2_O 3–5 times, and frozen in liquid nitrogen after removing water with clean filter paper.

The DNA extraction and Hi-C library construction were conducted by Biomarker Technologies (Beijing, China). In brief, nuclear DNA was extracted, digested with *DpnIII*, and biotinylated. Purified DNA was sheared to 300–700 bp fragments and used to construct the library and then sequenced by Illumina by 150-bp paired-end mode. Clean data was generated by filtering low-quality reads and removing adaptors and was mapped to the assembled ‘BS-40’ genome. The mapped data was used to group identification, sorting, orientation and finally assemble the chromosome-level genome of ‘BS-40’ with LACHESIS [59].

### Protein-coding gene prediction, non-coding RNA prediction, and gene function annotation

Repetitive sequences were predicted using LTR FINDER (v1.05), MITE-HUNTER, RepeatScout (v1.0.5), and PILER-DF (v2.4) to construct a primary repetitive sequences database of BS-40. The primary database was classified by PASTEClassifier and merged with the RepBase database as the final repetitive sequences database of ‘BS-40’. RepeatMasker (v4.0.6) [60] was used to identify repeat sequences based on the repetitive sequences database.

Protein-coding gene prediction was based on three strategies. For the *ab initio* gene prediction, Genscan, Augustus (v2.4), GlimmerHMM (v3.0.4), GeneID (v1.4), and SNAP were adopted to predict gene models. For homologous gene prediction, gene models were predicted by GeMoMa (v1.3.1) based on the proteins and mRNA sequences from *Arabidopsis thaliana*, *Vitis vinifera*, and *Oryza sativa*. For RNA-seq-based prediction, transcripts were assembled by Hisat (v2.0.4) and Stringtie (v1.2.3) based on the BS-40 genome, and then used to predict gene models with TransDecoder (v2.0) and GeneMarkS-T (v5.1) [61]. Finally, all gene models from the above three strategies were merged by EVM (v1.1.1) [62] to generate the consensus gene model of the BS-40 genome.

Non-coding RNAs were predicted by different approaches according to the characteristics of RNA structures. The micro-RNA and ribosomal RNAs were identified by Infenal 1.1 based on the miRbase and Rfam databases, respectively. The transfer RNAs were identified with tRNAscan-SE (v1.3.1) [63].

Predicted gene sequences were blasted to the NR, KOG, KEGG, and TrEMBL databases using BLAST (v2.2.31) [64] with an E-value threshold of 1e-5. The blast results from comparing to the NR database were annotated with the GO database by Blast2GO [65]. InterProScan (v5.8–49.0) [66] was used to annotate the motifs and domains information by aligning to the Pfam, PRINTS, ProDom, and SMART databases.

### Phylogenetic and comparative genomics analysis

The assembly statistics information of *Vitis* genomes were integrated from NCBI database. The genome and annotation files of *V. vinifera* (PN_T2T) were downloaded from the NCBI database (https://www.ncbi.nlm.nih.gov/datasets/genome/GCF_030704535.1/) [31]. The files of *V. vinifera* cv. Cabernet Sauvignon and *V. pseudoreticulata* were also downloaded from the NCBI database. The files of *V. amurensis* were downloaded from the TCMPG 2.0 database with number TCMPG20325 (https://cbcb.cdutcm.edu.cn/TCMPG2/genome/details/?id=TCMPG20325) [41]. The genome and annotation files of American *Vitis* species (*V. acerifolia*, *V. aestivalis*, *V. arizonica*, *V. berlandieri*, *V. girdiana*, *V. monticola*, *V. mustangensis*, *V. ripara*, *V. rotundifolia*, and *V. rupestris*) were released from a previous work [44].

The genomic synteny analysis was carried out by MCscan [67]. The subprograms of MCscan jcvi.formats.gff and jcvi.formats.fasta were used to convert the GFF annotation files to BED files, and convert the FASTA file of *V. vinifera* cv. PN40024 to CDS file, respectively. The homologous regions were identified and aligned using jcvi.compara.catalog ortholog. Then the paired synteny and macrosynteny were visualized with jcvi.compara.synteny and jcvi.graphics.karyotype, respectively [46]. Orthofinder (v2.3.7) was used to cluster the protein sequences of *V. piasezkii* var. *pagnucii* and these *Vitis* genomes [68]. Generated gene families were annotated with PANTHER V15 database. To study the evolutionary relationship between species, a maximum likelihood phylogenetic tree was constructed based on the single-copy protein sequences by IQ-TREE (v1.6.11) with JTT + F + I + R2 model. Divergence times of these species were calculated using the MCMCtree package of PAML (v4.9i) [69]. Based on the evolutionary tree with divergence times, the expansion and contraction of the gene families were defined using CAFE (v4.2) [70]. The significant expansion and contraction were determined by likelihood ratio tests (*P* < 0.05). The GO and KEGG enrichment were performed with clusterProfiler.

### NLR genes annotation

The amino acid sequences of NLR genes in the ‘BS-40’ genome were annotated first using NLR-annotator. In addition, InterProScan was used to annotate the protein domains of these NLR genes. These results were merged to obtain a comprehensive set of NLR genes of ‘BS-40’. The maximum-likelihood tree of NLR genes was constructed with IQ-TREE using VT + F + R8 model selected by ModelFinder [71]. To analyze the presence and absence of these NLR genes, we choose GMAP to predict the homologs of NLR genes in ‘BS-40’ among other 14 *Vitis* genomes [72]. The NLR genes located at a distance of less than 200 kb and fewer than eight non-NLR genes were defined as a gene cluster [73].

### Transcription level analysis of candidate resistance genes

The leaves of ‘BS-40’ and ‘Cabernet Sauvignon’ at 0, 12, 24, 48, and 72 h postinoculation (hpi) with *En.* NAFU1 were collected to perform RNA-seq. Total RNA was extracted using the RNAprep Pure Plant Plus Kit (TIANGEN, Beijing, China). The RNA-seq libraries were generated by NEBNext® Ultra™ RNA Library Prep Kit for Illumina® (NEB, USA). Library quality was assessed on an Agilent Bioanalyzer 2100 system (Agilent, USA). The qualified libraries were sequenced on an Illumina NovaSeq 6000 platform. Low-quality reads were filtered and clean reads were mapped to the assembled ‘BS-40’ genome using hisat2 (v2.2.1) [74]. The expression levels were processed with featureCounts (v2.0.3) and the differentially expressed genes (DEGs) were identified using DESeq2 (v1.38.2) with *P*adj <0.05 and |log2FoldChange| >1.

### Powdery mildew resistance identification

The grapevine leaf resistance to powdery mildew of the population was processed according to the quick evaluation method [45] and in natural conditions [75]. The resistance to powdery mildew of ‘BS-40’, ‘PC’, and their progenies were identified four times from 2020 to 2022 in three ways. Firstly, the conidia suspension (10^5^ conidia/mL in distilled water containing 0.001% Tween 20) of the powdery mildew isolate *En.* NAFU1 was sprayed on the young leaves in the greenhouse (2020.08). The resistance was observed 14 days post inoculation. While the leaves of ‘BS-40’ and ‘PC’ were stained with trypan blue and Diaminobenzidine (DAB) at 24, 48, 72, 96, and 120 hpi to observe the conidia and hyphae structure and cell death. Secondly, the leaves without inoculation were observed in natural conditions (2020.11). Thirdly, the detached young leaves were inoculated with the conidia suspension of *En.* NAFU1 (2021.04 and 2022.05). All hybrids were stained with trypan blue and DAB at 72 hpi, while ‘BS-40’ and ‘PC’ were observed at 24, 48, 72, 96, and 120 hpi again. These stained results were observed with an Olympus BX51 microscope.

For the greenhouse leaves, the resistance levels were classified to HR (0%–5%), R (5%–25%), S (25%–50%), and HS (50%–100%) according to the percentages of lesions over the whole leaf area. For the stained leaves, the resistance levels were classified to HR (0%–40%), R (40%–60%), S (60%–80%), and HS (80%–100%) according to the appressoria rate. At 72 hpi, the length of the longest hyphae germinated from a single conidium was measured as the hyphae length with 30 replicates. And the newly formed conidia from the end of hyphae were counted in 1 mm^2^ leaves with 3 replicates. The most stable resistance level of hybrids was used for statistics. And these samples with stable resistance levels and well growth conditions were used in GWAS analysis (Table S14).

### Genome resequencing and GWAS

Young leaves of ‘PC’ and its F_1_ progenies with ‘BS-40’ were collected in the spring of 2021, and frozen in liquid nitrogen immediately. The genomic DNAs extracted following a standard cetyltrimethylammonium bromide (CTAB) protocol, were used to construct the library according to the Illumina library preparation protocol with an insert size of 350 bp and sequenced on the Illumina Hiseq platform by paired-end in 150-bp mode. The adaptors and low-quality sequences of raw reads were trimmed to obtain clean reads. All sequencing procedures were carried out by Annoroad, Zhejiang, China.

Clean reads of re-sequenced samples were mapped to the assembled genome of BS-40, de-duplicates, and realigned by Sentieon [76] and generated SNPs. These SNPs were filtered under the condition: —indep 50 5 2—maf 0.05—geno 0.1—hwe 0.001 by PLINK [77]. PCA was carried out to verify the influence on progenies of ‘PC’ when ‘BS-40’ as the male parent. The GWAS analysis was conducted by PLINK with the standard case/control method of logistic model. The Manhattan plot and the Quantile-Quantile plot (Q-Q plot) were generated by R package qqman.

### Population selection and QTL analysis

The resistant F_1_ progenies were regarded as a resistant population, while the susceptible F_1_ progenies with the female parent ‘PC’ were regarded as a susceptible population. The genome-wide distribution of population fixation statistics *F*_ST_ and the nucleotide diversity *π* [46] were estimated for each 50 kb sliding window with a step size of 25 kb (windows with fewer than five SNPs were discarded). Candidate selective regions with both top 1% of *F*_ST_ and log_2_(*π*_R/*π*_S) were extracted to identify the genomic regions with high differentiation and different nucleotide diversity between the resistant and susceptible population.

The same SNP dataset and phenotype file were used for QTL analysis using R/qtl package [78]. The genotypes 0/0 and 1/1 were converted to A and B, respectively, while 0/1 and 1/0 were converted to H. The conditional genotype probabilities were calculated with 1 cM density and 0.001 genotyping error rate using the R/qtl function *calc.genoprob*. The Haley-Knott regression of Sigle-QTL analysis was performed using the R/qtl function *scanone*. The LOD thresholds were determined based on 1000 permutations (*P* = 0.05).

### Recombinants analysis

Fifteen SNPs highly associated with GPM resistance around locus *Ren17* were selected and designed as markers based on the re-sequencing data and the GWAS results. These SNPs were heterozygous in ‘BS-40’ but homozygous in ‘PC’ (Table S18). SNPs of all progenies around these markers were screened. Regions with more than 50% SNPs were heterozygous in susceptible progenies were regarded as recombined from ‘BS-40’, and these regions with more than 50% SNPs were homozygous in resistant progenies were considered recombined from ‘PC’ vice versa.

### The development and genotyping of dCAPS marker

The dCAPS primer was designed by dCAPS Finder 2.0 based on the genome sequence and SNP polymorphism [79]. The genomic DNA of ‘BS-40’, ‘PC’, and their progenies were extracted following a standard CTAB protocol. PCR amplifications were performed using 2x Rapid Taq Master Mix (Vazyme Bio Co, Nanjing, China). The amplification program consists of an initial denaturation at 95°C for 5 min, followed by 35 cycles of denaturation at 95°C for 30 s, annealing at 56°C for 30 s, and elongation at 72°C for 5 s, and finished with a final elongation at 72°C for 5 min. PCR products were digested with *Sma* I (Takara Bio, Beijing, China) following the manufacturer’s protocol. The digestion products were separated on 8% polyacrylamide gel electrophoresis.

## Supplementary Material

Web_Material_uhaf146

## Data Availability

The genome sequence of ‘BS-40’ has been submitted to the National Center for Biotechnology Information (BioProject ID: PRJNA1104008, accession number: JBDIVC000000000). The raw sequences of the transcriptome of ‘BS-40’ and ‘CS’ were submitted to the SRA database (BioProject ID: PRJNA1105926). The resequencing sequences of ‘PC’ and its’ progenies crossed with ‘BS-40′ were submitted to the SRA database (BioProject ID: PRJNA1105918).
