# Supplementary material for: Chromosome-level reference genome of Vitis piasezkii var. pagnucii provides insights into a new locus of resistance to grapevine powdery mildew
Source: Hortic Res. 2025 Jun 10;12(9):uhaf146. doi: 10.1093/hr/uhaf146 (PMC12313342; doi:10.1093/hr/uhaf146)
Supplement: Web_Material_uhaf146 [file web_material_uhaf146.zip › Supplemental Figures.pdf]

**Supplementary Figures for**

# Chromosome-Level Reference Genome of *Vitis piasezkii* var. *pagnucii* provides insights into a new locus of resistance to grapevine powdery mildew.

**Liang Zhao[1, 2], Yang Hu[1, 2], Qian-Yu Ji[1, 2], Li-Xue Gong[1, 2], Meng-Jiao Lu[1, 2], Xue-Na Yu[1, 2], Zhuo-Shuai Jin[1, 2], Min Zhou[1, 2], Xue-Lei Dai[3], Shun-Yuan Xiao[4], Yu Jiang[3, *], Ying-Qiang Wen[1, 2, *]**

[1]State Key Laboratory of Crop Stress Resistance and High-Efficiency Production, College of Horticulture, Northwest A&F University, Yangling Shaanxi 712100, China

[2]Key Laboratory of Horticultural Plant Biology and Germplasm Innovation in Northwest China, Ministry of Agriculture and Rural Affairs, Yangling Shaanxi 712100, China

[3]Key Laboratory of Animal Genetics, Breeding and Reproduction of Shaanxi Province, College of Animal Science and Technology, Northwest A&F University, Yangling Shaanxi 712100, China

[4]Department of Plant Science and Landscape Architecture, Institute for Bioscience and Biotechnology Research, University of Maryland, Rockville, Maryland, USA

***Correspondence author**

Ying-Qiang Wen: wenyq@nwsuaf.edu.cn

Yu Jiang: yu.jiang@nwafu.edu.cn

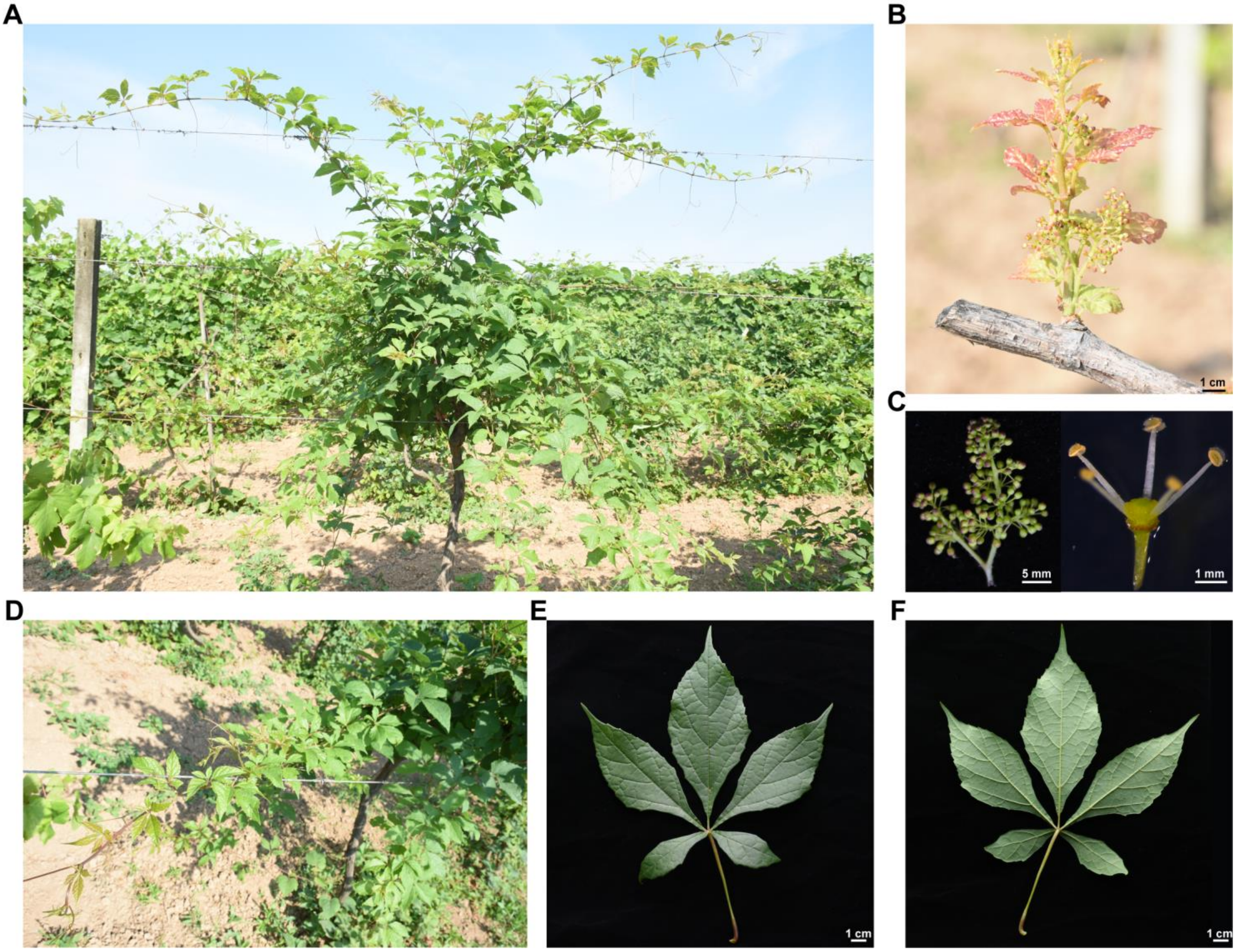


**Figure S1. Plant Morphology of *Vitis piasezkii* 'BS-40'. (A)** *Vitis piasezkii* 'BS-40' used in this study. **(B)** The young shoot, **(C)** flowers, and **(D)** mature shoot of 'BS-40'. **(E)** The adaxial side, and **(F)** the abaxial side of 'BS-40' mature leaves.

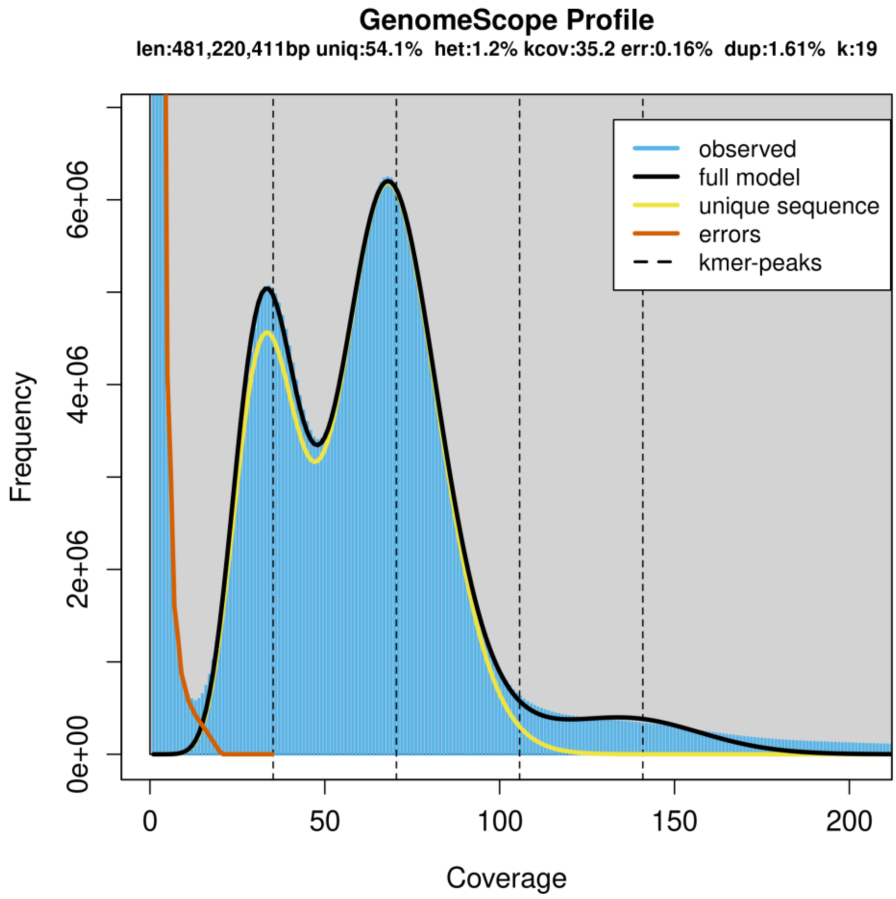


**Figure S2. The GenomeScope k-mer profile plot of the 'BS-40' genome survey.** The statistics of genome size (len), heterozygosity levels (het), duplication rates (dup), and error rates (err) were shown.

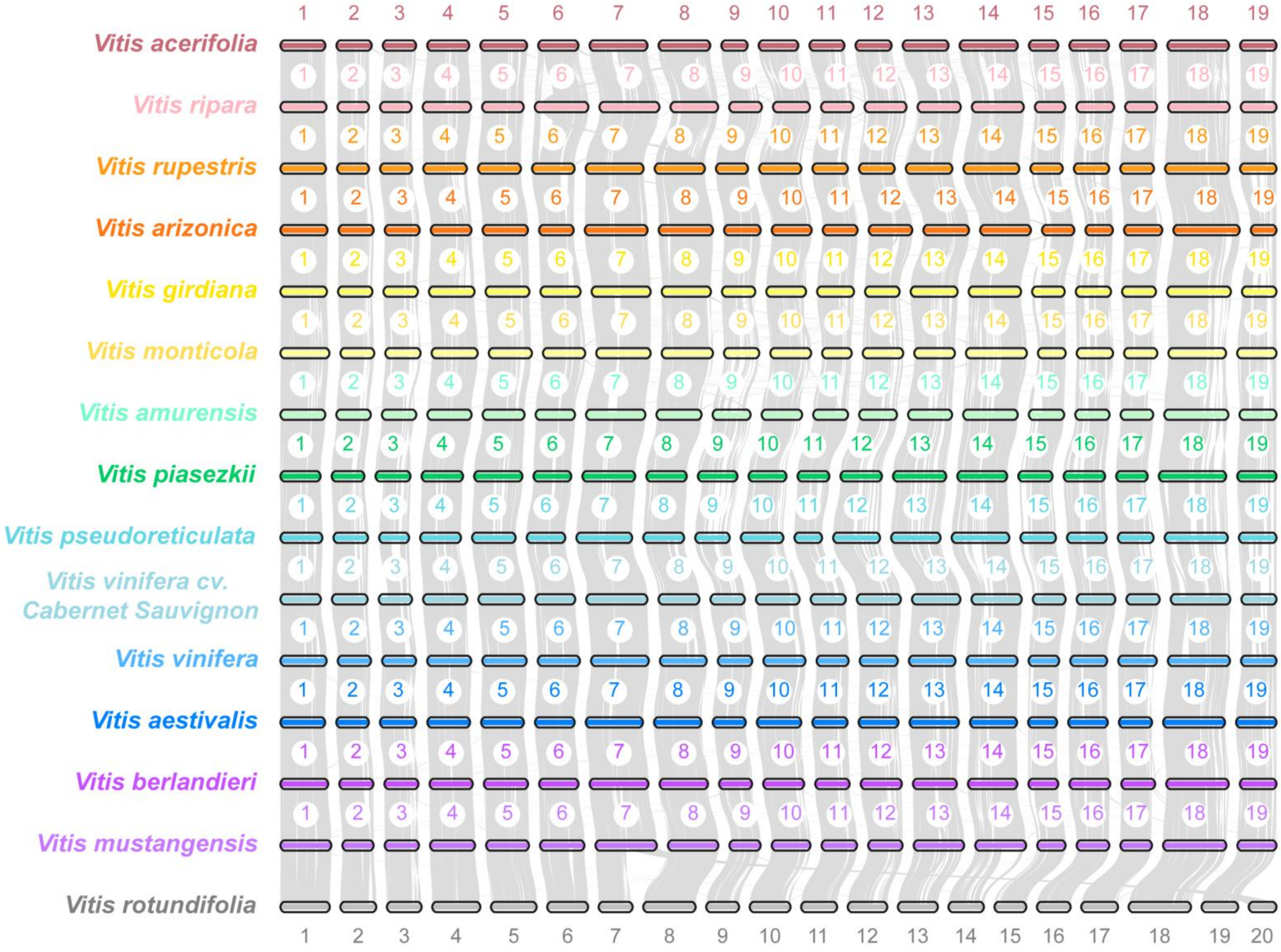


**Figure S3. Synteny analysis of 15 grapevine genomes.** The left panel displays the names of 15 *Vitis* genomes. The numbers indicate chromosome identifiers. Gray lines connected homologous syntenic regions among these genomes.

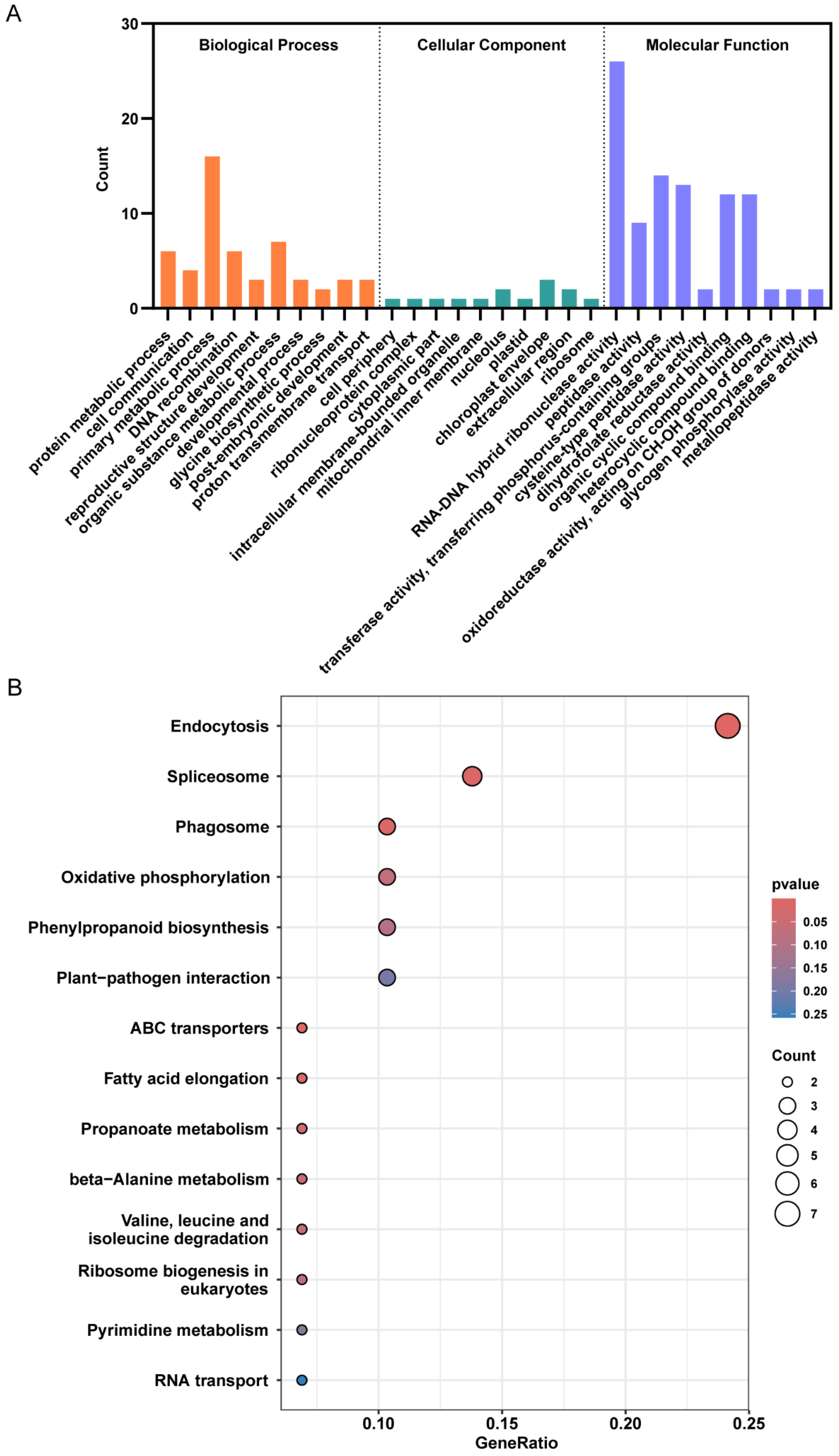


**Figure S4. Enrichment of the unique genes in ‘BS-40’** **(A)** Top 10 enriched GO terms in biological process, cellular component, and molecular function for ‘BS-40’-unique genes. **(B)** Significantly enriched KEGG pathways identified for ‘BS-40’-unique genes.

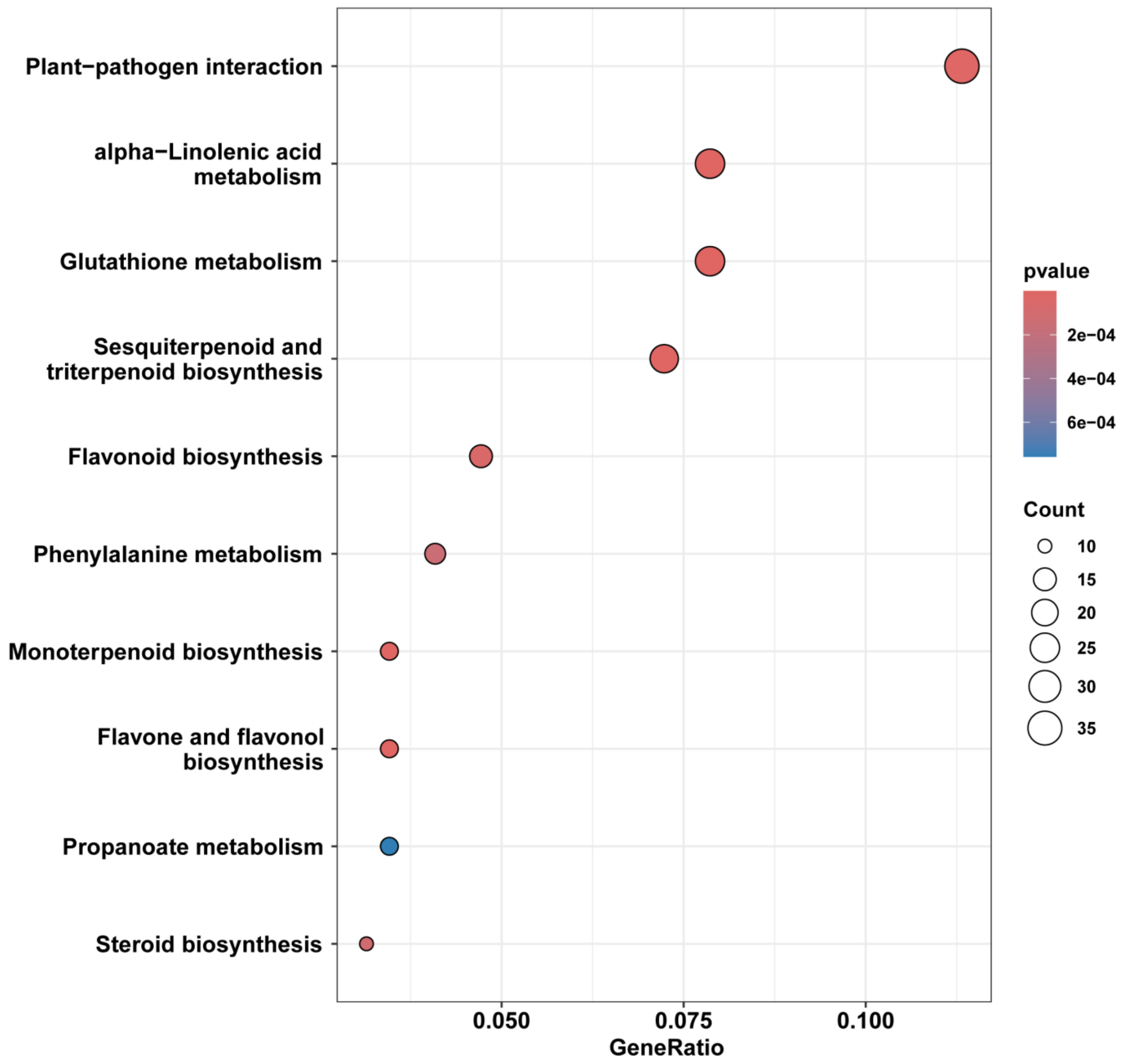


**Figure S5. Top 10 significant KEGG pathways enriched in the expanded gene families of ‘BS-40’.**

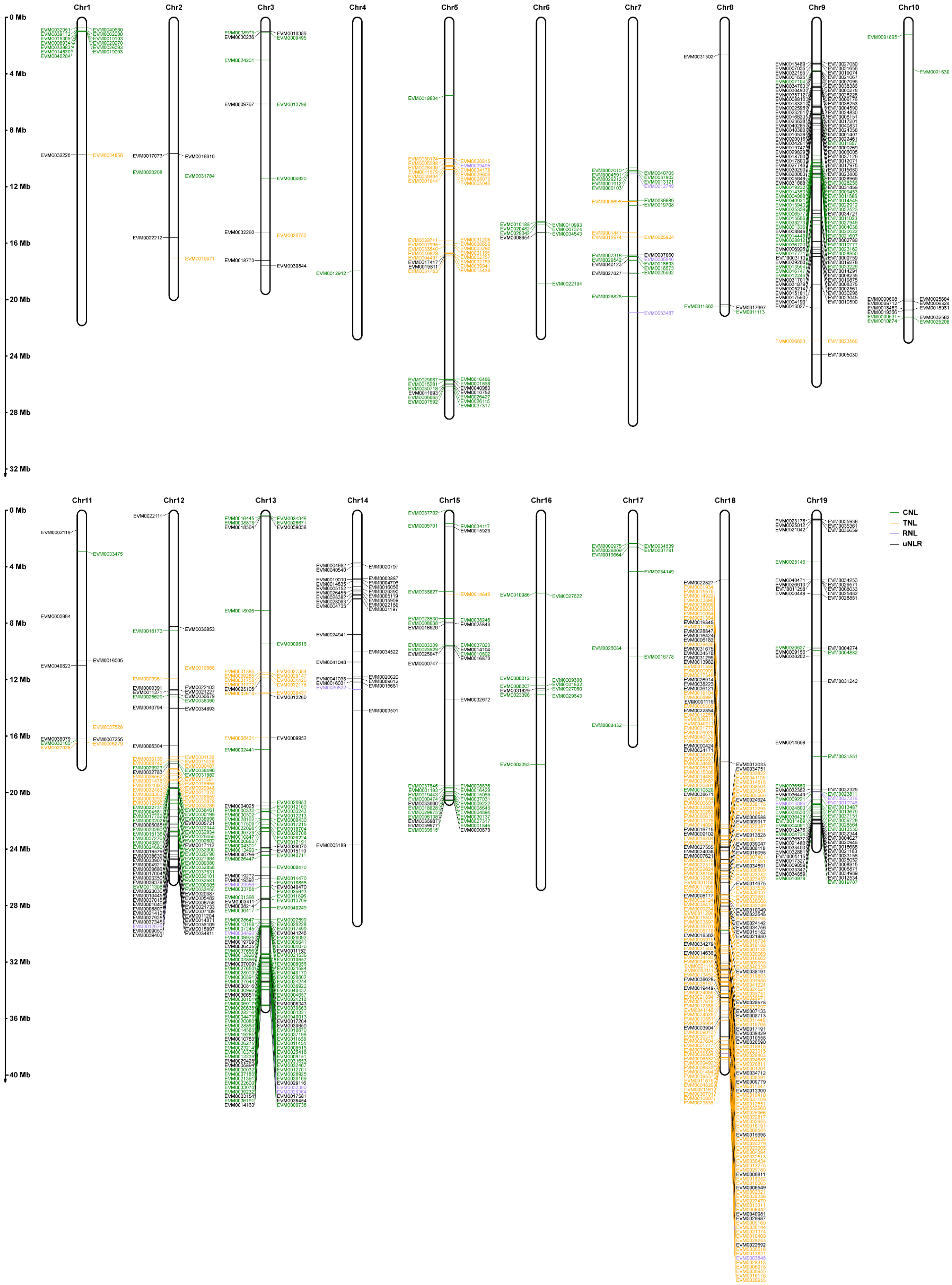


**Figure S6. The distribution of 897 NLR resistance genes in all chromosomes of the 'BS-40' genome.** Green bars represent CNLs (306), orange bars represent TNLs (242), purple bars represent RNLs (14), black bars represent uncharacteristic NLRs (335).

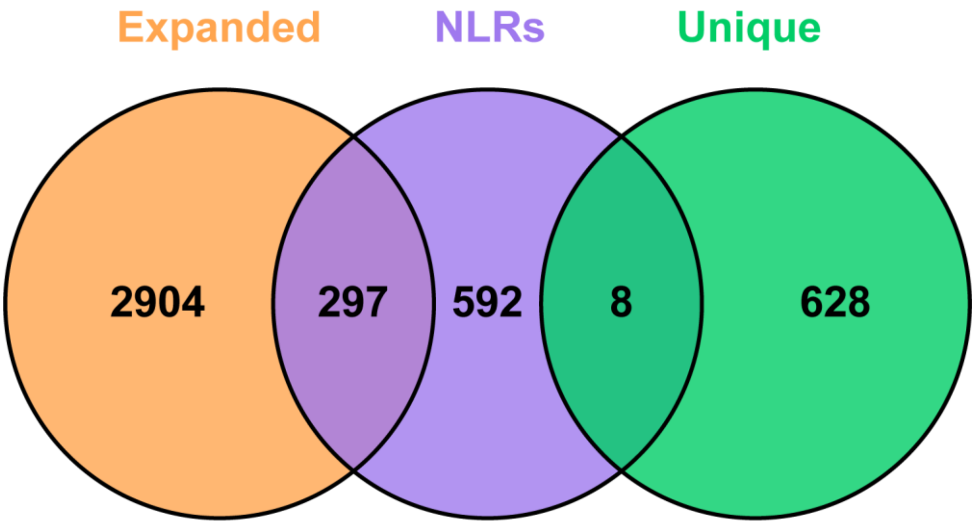


**Figure S7 . The relationship of the unique, expanded, and NLR genes of the 'BS-40' genome.** Green represents unique genes of 'BS-40' among 15 *Vitis* genomes, orange denotes expanded genes identified through phylogenetic analysis, and purple indicates NLR genes obtained by gene annotation. Black numbers indicate gene counts.

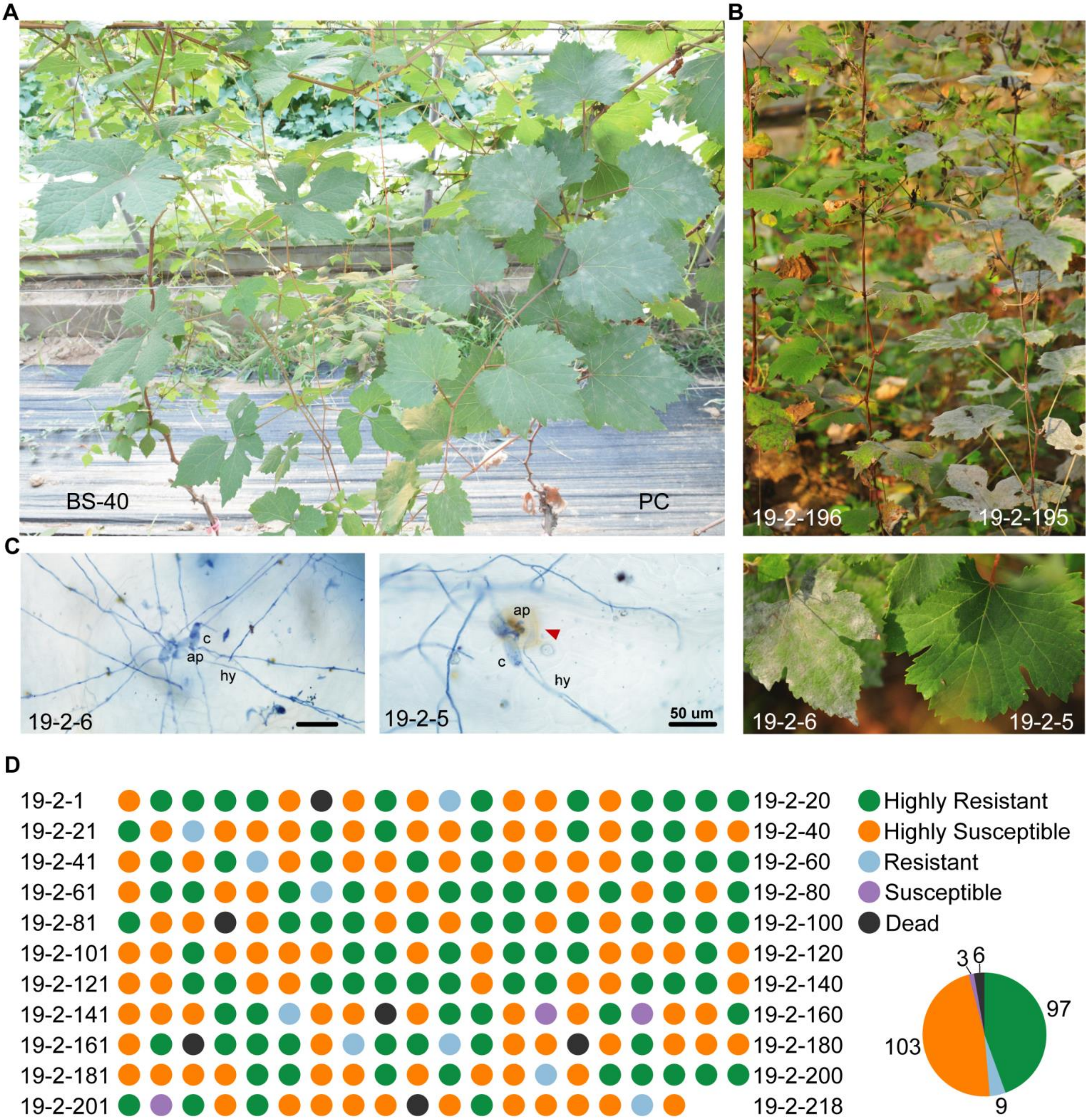


**Figure S8. The phenotype and statistics of powdery mildew resistance of crossing progenies from 'PC' × 'BS-40'. (A)** The incidence of GPM of 'BS-40' and 'PC' under natural conditions. **(B)** Different $F_1$ individuals of 'PC' × 'BS-40' at 14 days post inoculation with powdery mildew *En. NAFU1* in the field. **(C)** The staining of $F_1$ individuals detached leaves at 3 days post inoculation with *En. NAFU1*. The conidium (c), appressoria (ap), and hyphae (hy) of *En. NAFU1* were stained blue. The red triangle pointed to the $H_2O_2$ accumulation in the cell death progress. **(D)** Statistics of powdery mildew resistance level of 218 crossing progenies of 'PC' × 'BS-40'.

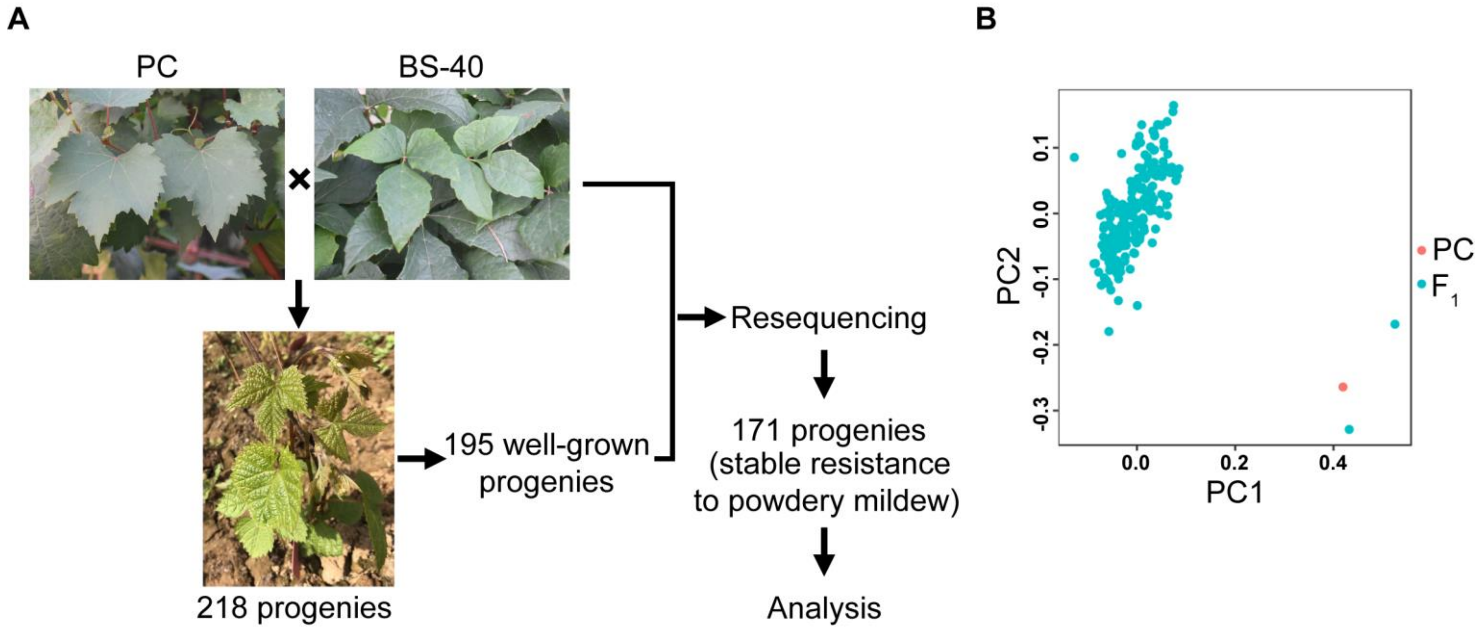


**Figure S9. The scheme and the principal component analysis (PCA) analysis of genome resequencing of crossing progenies of ‘PC’ × ‘BS-40’. (A)** The scheme of genome resequencing of 171 progenies (with both compound leaves and pentagonal leaves) crossed by ‘PC’ (pentagonal leaves) and ‘BS-40’ (compound leaves). **(B)** The PCA of SNPs generated from re-sequenced $F_1$ progenies and ‘PC’ mapped to the ‘BS-40’ genome.

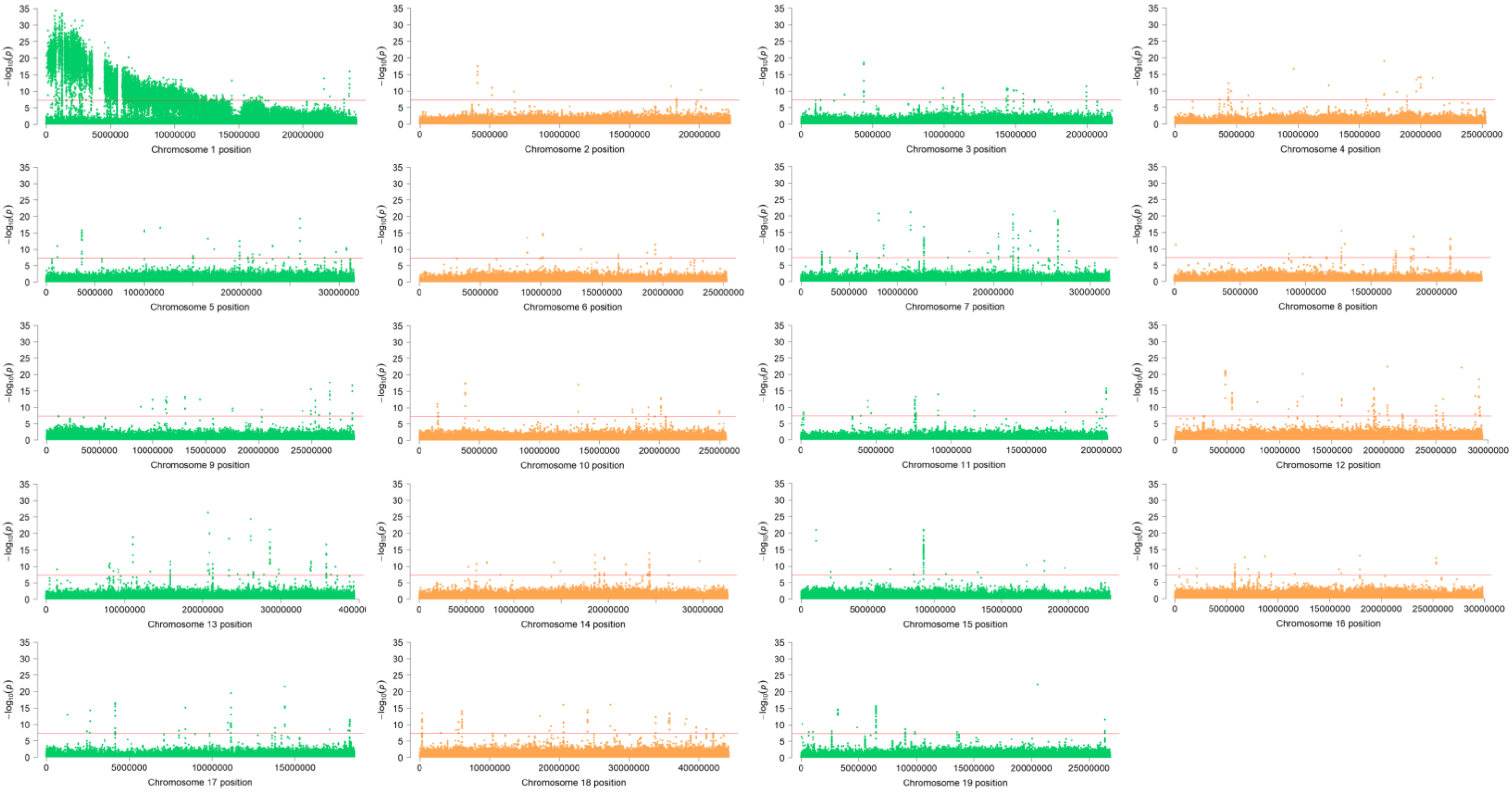


**Figure S10. The GWAS results of 19 chromosomes.** The red horizontal line in manhattan plot depicts the significance threshold (-log10 ($p$) = 7.5).

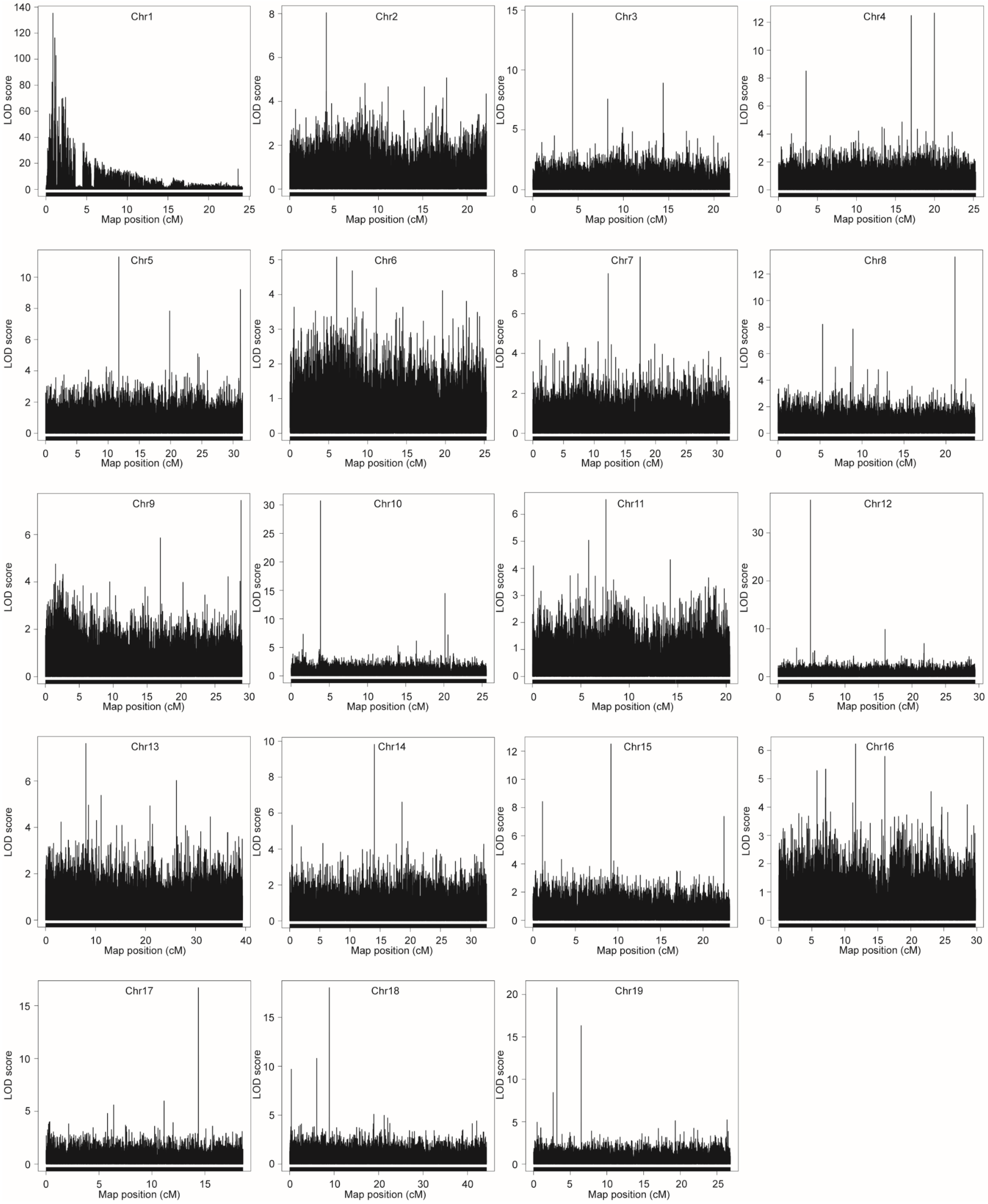


**Figure S11. The QTL results of 171 $F_1$ population of 'PC' and 'BS-40'.** The Haley-Knott regression of Sigle-QTL analysis was performed and the LOD thresholds were determined based on 1000 permutations (*P* = 0.05).

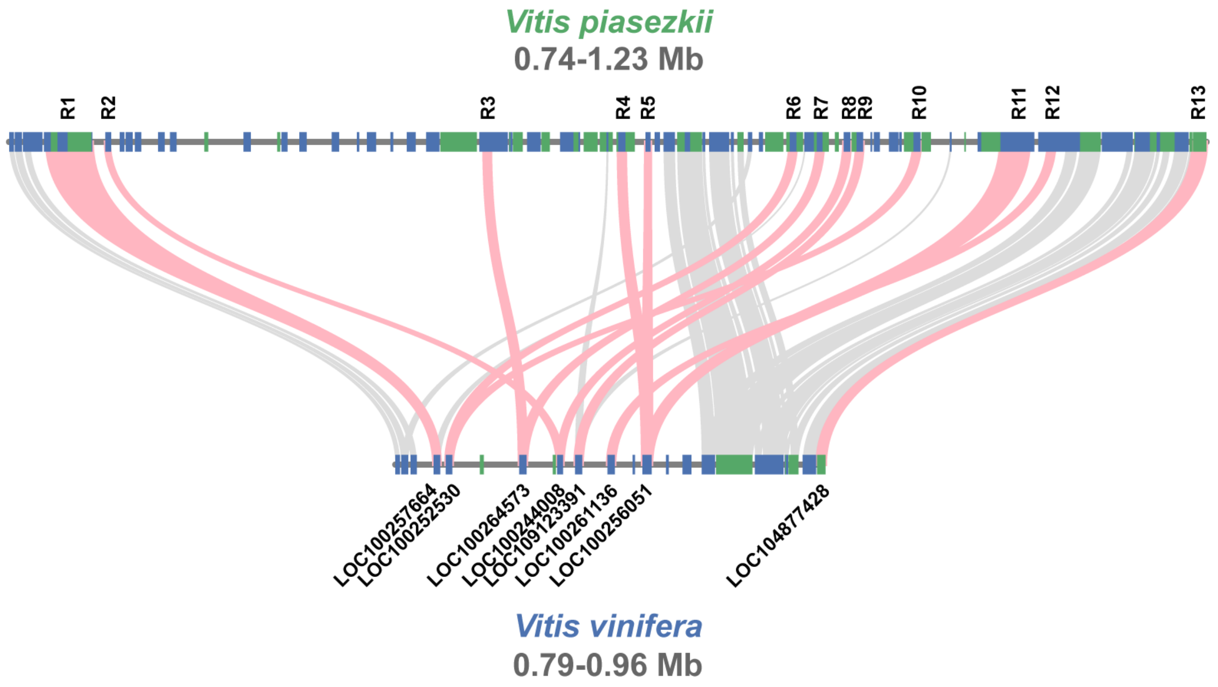


**Figure S12. The gene-level synteny analysis of the *Ren17* locus between 'BS-40' and *V. vinifera* cv. PN40024.** Blue rectangles represent exons, green rectangles indicate introns, and gray straight lines denote intergenic regions. R1 – R13 correspond to the 13 NLRs genes within the *Ren17* locus of 'Baishui-40'. Pink lines illustrate synteny relationships between these genes and their homologous in PN40024.

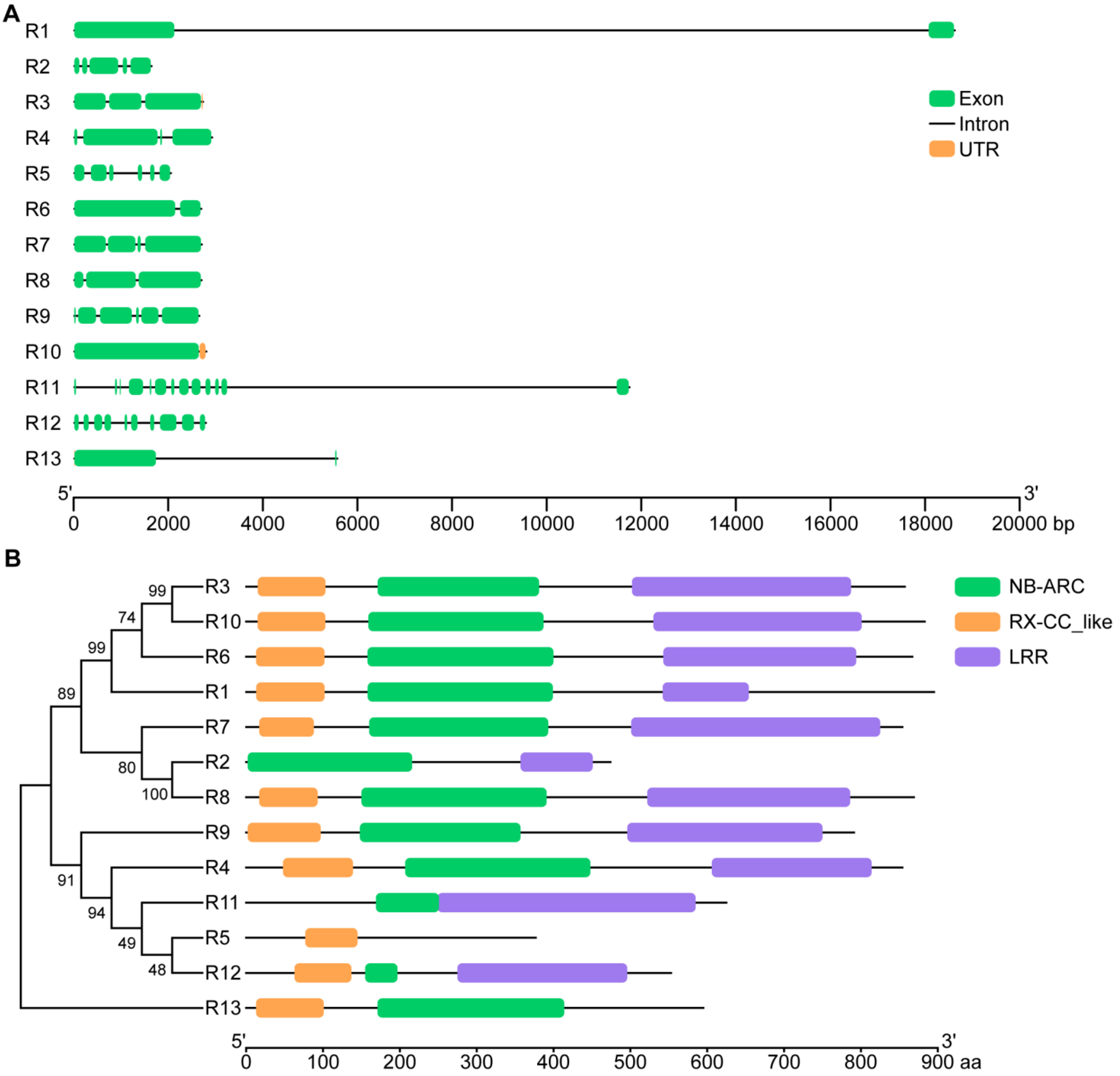


**Figure S13. The features of 13 resistant genes in the *Ren17* locus.**
**(A)** The position of exons in 13 resistant genes. **(B)** Phylogenetic analysis and conserved domains in protein sequences of 13 resistance genes in *Ren17* locus.

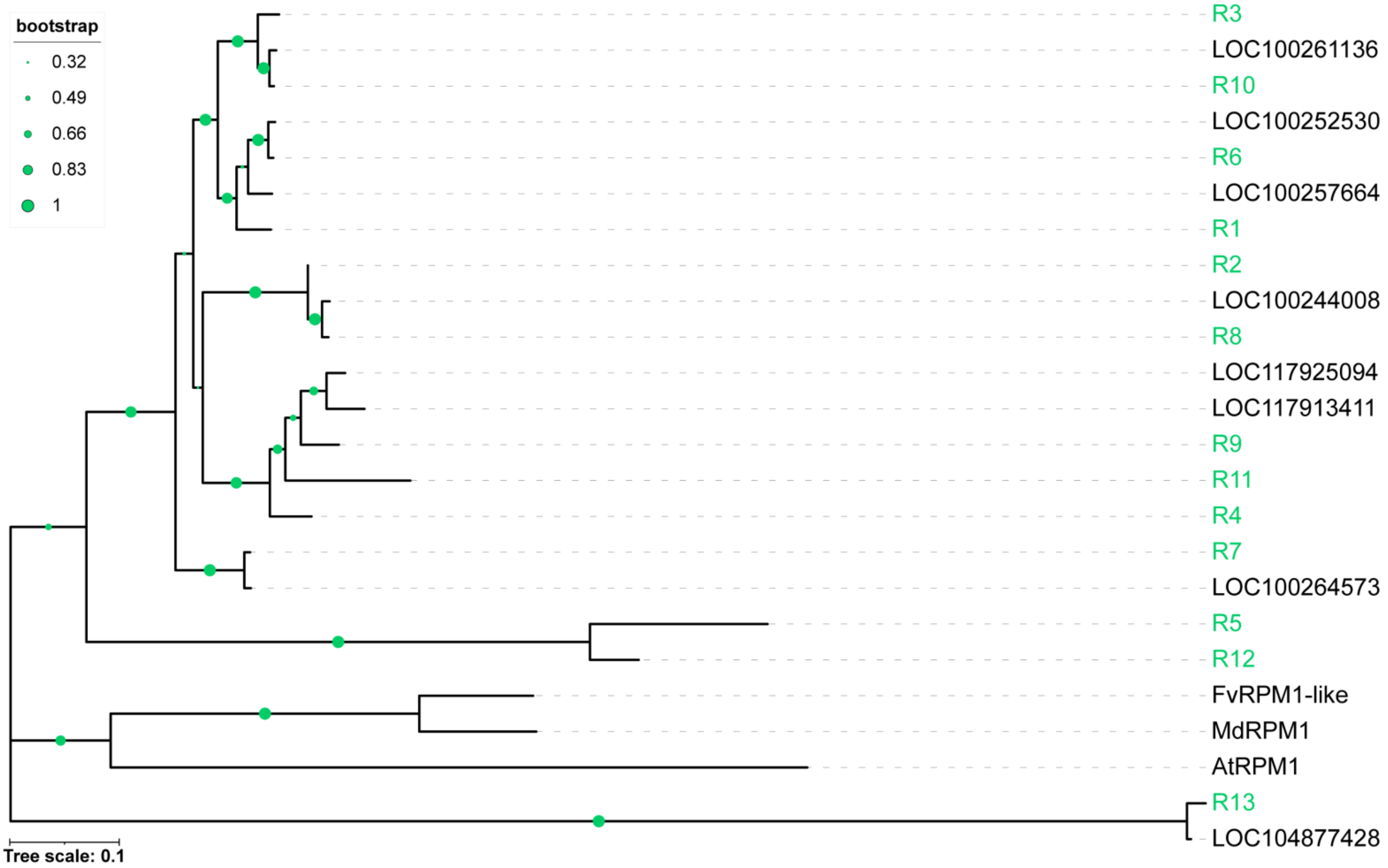


**Figure S14. A phylogenetic tree of resistance genes in the *Ren17* locus with their homologous genes in *Vitis* species and three other plants.**

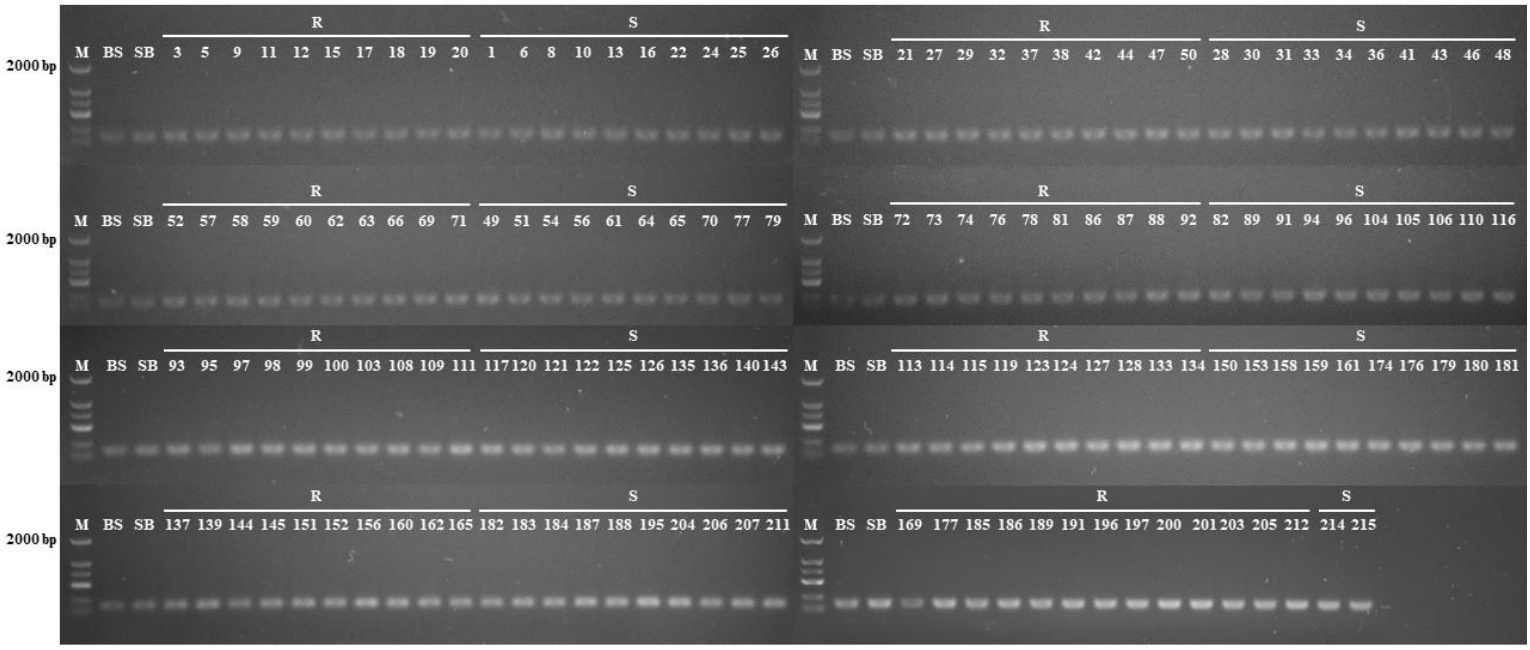

**Figure S15. The PCR products of ‘BS-40’, ‘PC’, and their progenies using the dCAPS primer pair of SNP chr1_1066968.**
